# An exploratory single-cell analysis of peripheral blood mononuclear cells from vedolizumab-treated Crohn’s disease patients identifies response-associated differences among the plasmacytoid dendritic cells and classical monocytes

**DOI:** 10.3389/fimmu.2025.1551017

**Published:** 2025-08-15

**Authors:** Andrew Yung Fong Li Yim, Ishtu Hageman, Vincent W. Joustra, Ahmed M. I. M. Elfiky, Mohammed Ghiboub, Evgeni Levin, Jan Verhoeff, Caroline Verseijden, Iris Admiraal, Manon de Krijger, Manon E. Wildenberg, Marcel Mannens, Marja E. Jakobs, Susan B. Kenter, Alex T. Adams, Jack Satsangi, Geert R. D’Haens, Wouter J. de Jonge, Peter Henneman

**Affiliations:** ^1^ Tytgat Institute for Liver and Intestinal Research, Amsterdam University Medical Center (UMC) location University of Amsterdam, Amsterdam, Netherlands; ^2^ Amsterdam Gastroenterology Endocrinology Metabolism, Amsterdam, Netherlands; ^3^ Department of Gastroenterology and Hepatology, Amsterdam University Medical Center (UMC) location University of Amsterdam, Amsterdam, Netherlands; ^4^ Emma Children’s Hospital, Pediatric Surgery, Amsterdam University Medical Center (UMC) location University of Amsterdam, Amsterdam, Netherlands; ^5^ Genome Diagnostics Laboratory, Department of Human Genetics, Amsterdam University Medical Center (UMC) location University of Amsterdam, Amsterdam, Netherlands; ^6^ Amsterdam Reproduction and Development, Amsterdam, Netherlands; ^7^ Amsterdam Infection & Immunity, Amsterdam, Netherlands; ^8^ Center for Translational Immunology, University Medical Centre Utrecht, Utrecht University, Utrecht, Netherlands; ^9^ Horaizon BV, Delft, Netherlands; ^10^ Department of Molecular Cell Biology and Immunology, Amsterdam University Medical Center (UMC) location Free University Amsterdam, Amsterdam, Netherlands; ^11^ Cancer Center Amsterdam, Amsterdam, Netherlands; ^12^ Translational Gastroenterology Unit, John Radcliffe Hospital, Oxford, United Kingdom; ^13^ Department of Surgery, University of Bonn, Bonn, Germany

**Keywords:** single-cell RNA-sequencing, scRNAseq, cytometry by time of flight, cyTOF, vedolizumab, therapy response, T cell, pDC

## Abstract

**Background:**

Vedolizumab (VDZ) is a monoclonal antibody approved for the treatment of Crohn’s disease (CD). Despite its efficacy, non-response to VDZ is common in clinical practice with no clear understanding of how it manifests. Here, we performed an exploratory study characterizing the cellular repertoire of responders and non-responders to VDZ during treatment.

**Methods:**

Peripheral blood mononuclear cells (PBMCs) were isolated from CD patients on VDZ treatment that were either steroid-free responder (N = 4) or non-responder (N = 4). Response was defined as ≥3 drop in Simple Endoscopic Score for Crohn’s Disease (SES-CD) in combination with a ≥50% reduction in C-reactive protein (CRP) and fecal calprotectin and/or a ≥3 point drop in Harvey-Bradshaw Index (HBI). Single-cell repertoires were characterized using single-cell RNA-sequencing (scRNAseq) and mass cytometry by time of flight (CyTOF).

**Results:**

Non-responders to VDZ presented more T cells, but fewer myeloid cells, with plasmacytoid dendritic cells (pDCs) being the most notably lower among non-responders. At a transcriptional level we observed that T-cell expression of genes involved in for Toll-like receptor (TLR), NOD-like receptor (NLR), and mitogen-activated protein kinases (MAPK) signaling pathways were decreased among non-responders. Similarly, non-responder-derived classical monocytes presented lower expression of genes involved in cytokine-cytokine receptor signaling.

**Conclusions:**

Non-response to VDZ during treatment is associated with differences in abundance and expression among T and myeloid cells.

## 1 Introduction

Crohn’s disease (CD) and ulcerative colitis (UC) are incurable, chronic, inflammatory conditions of the gastrointestinal tract characterized by a relapsing-remitting transmural inflammation of the digestive tract belonging to the family of inflammatory bowel diseases (IBD). Current treatments for CD and UC include the use of monoclonal antibodies that target mediators of inflammation with the goal of ameliorating the inflammatory phenotype and/or maintaining a state of clinical and endoscopic remission. One such monoclonal antibody is vedolizumab (VDZ), which was approved for use in patients with CD in 2014 by the United States Food and Drug Administration as well as the European Medicines Agency (1).

VDZ targets the gut homing receptor complex integrin α_4_β_7_ (also known as lymphocyte Peyer’s patch adhesion molecule 1; LPAM-1) (2, 3), which prevents it from binding mucosal vascular addressin cell adhesion molecule 1 (MAdCAM-1), a molecule expressed exclusively by the intestinal endothelial cells. By preventing integrin α_4_β_7_ from binding MAdCAM-1, the attachment and stabilization of circulating immune cells that express integrin α_4_β_7_ to high endothelial venules in the gut is destabilized, thereby abrogating gut-homing capabilities (4–6). While VDZ has traditionally been discussed within the context of the T cell lineage (7–10), more recent studies suggest that the myeloid (11, 12) as well as B cells (13) are affected by VDZ treatment as well. Despite the advances VDZ therapy has provided patient care, the efficacy or therapy response rate is reported to be approximately between 30% to 45% (1, 14–16) with a recent meta-analysis indicating that loss of response towards VDZ among CD patients was estimated at 47.9 per 100 person-years (17). To date, we have no proper understanding why only a subgroup of patient responds to therapy, nor do we have a prognostic biomarker for predicting response to VDZ therapy. To better understand how response to VDZ manifests, we conducted an exploratory case-control study to characterize the immune cell composition of peripheral blood mononuclear cells (PBMCs) from CD patients on VDZ treatment. Specifically, we compared responders with non-responders using single-cell RNA-sequencing (scRNAseq) and cytometry by time of flight (CyTOF).

## 2 Materials and methods

### 2.1 Cohort assembly and sample collection

Patients included were obtained from the EPIC-CD study, which is a multi-center consortium with the goal of identifying prognostic biomarkers at the level of peripheral blood (PBL) DNA methylation capable of predicting response to adalimumab, infliximab, VDZ, and ustekinumab prior to treatment in CD patients (18). Adult CD patients were included if they presented with active ulcerative disease as defined by a simple endoscopic score CD (SES-CD) >3. Patients were excluded if they presented with ongoing malignancy, concomitant inflammatory diseases, no measurable serum drug concentration, presence of anti-VDZ antibodies, or stopped treatment in the absence of response assessment. For the current study, 8 VDZ-treated CD patients (4 responders and 4 non-responders) were sampled for PBL at a median of 41 weeks (interquartile range 31-70) into treatment during routine care at the AmsterdamUMC hospital, location AMC, Amsterdam, Netherlands between 2018 and 2019 (**Table 1**). VDZ infusions of 300mg were provided at week 0, 2 and 6 followed by infusions every eight weeks. Response was assessed at approximately 41 weeks based on a reduction relative to the baseline measurement in endoscopic (ΔSES-CD≥50%) in combination with either clinical- (ΔHarvey Bradshaw Index (HBI)≥3 or HBI≤4) and/or biochemical (ΔC-reactive protein (CRP)≥50% or CRP≤5 g/mL or Δfecal calprotectin (FCP)≥50% or FCP≤250 µg/g) criteria in the absence of systemic corticosteroids. Immediately after collecting peripheral blood, peripheral blood mononuclear cells (PBMC) were isolated by means of Ficoll (GE Healthcare) separation and IMDM medium (Gibco) supplemented with 10% DMSO and 50% FBS (Serana). Isolated PBMCs were stored overnight at -80 C in Mr. Frosty freezing containers (Thermo) where after they were transferred to liquid nitrogen until cohort completion.

**Table 1:** Patient characteristics at time of sampling included in the scRNAseq and CyTOF.

	Responders (N = 4)	Non-responders (N = 4)	p-value
Female, N (%)	3 (75)	4 (100)	1.00
Age, years, median (IQR)	40 (35-48)	48 (36.5-61.25)	0.77
Disease duration [years], median (IQR)	10.5 (7.5-18.5)	7 (4.5-23)	
Ethnic background, N (%)			
Caucasian	3 (75)	3 (75)	1.00
Duration between sampling and treatment start [weeks], median (IQR)	50.6 (45.7-71.4)	29.2 (13.2-44.8)	0.25
T2 VDZ trough concentration [μg/mL], median (IQR)	17.5 (11.8-26)	26 (2.7-28.3)	0.68
SES-CD, median (IQR)			
T1	5.5 (5-8)	6 (6-6.5)	0.58
T2	1.5 (0-4)	3 (1.5-3.5)	1.00
HBI, median (IQR)			
T1	9.5 (6-14.5)	8 (8-9)	1.00
T2	2.5 (1-4.3)	9 (6.5-11.3)	0.11
CRP [mg/L], median (IQR)			
T1	9.8 (6.5-14.4)	2.2 (1.4-2.8)	0.03
T2	7.6 (5.5-8.9)	3.1 (0.8-5.9)	0.20
FCP [μg/g], median (IQR)			
T1	328.5 (168-849.5)	262.5 (189-305.3)	0.69
T2	54.5 (24.8-168)	442 (250.5-522)	0.23
Disease location, n (%)			
Ileal disease (L1)	3 (75)	1 (25)	0.49
Colonic disease (L2)	–	–	–
Ileocolonic disease (L3)	1 (25)	3 (75)	0.49
Disease behavior, N (%)			
Non structuring/penetrating (B1)	1 (25)	2 (50)	1.00
Structuring (B2)	2 (50)	2 (50)	1.00
Penetrating (B3)	1 (25)	–	1.00
Perianal disease (p)	1 (25)	–	1.00
Previous IBD-related surgery, N (%)	2 (50)	1 (25)	1.00
Concomitant medication, N (%)			
Immunomodulators	–	–	–
Prednisone	–	–	–
Previous treatment exposure, N (%)			
Immunomodulators	3 (75)	1 (25)	0.49
Anti-TNF (ADA or IFX)	1 (25)	1 (25)	1.00
Smoking, N (%)			
Never	1 (25)	1 (25)	1.00
Active	–	3 (75)	0.14
Former	3 (75)	–	0.14

Overview of the demographics of the included patients. P-values were calculated using either the Fisher exact test or the Mann Whitney U test for categorical and continuous variables, respectively. IQR: Interquartile range. T1: Start of treatment. T2: Response assessment. SES-CD: Simple endoscopic score for Crohn’s disease. HBI, Harvey Bradshaw Index; CRP, C-reactive protein; FCP, Fecal calprotectin; ADA, Adalimumab; IFX, Infliximab.

### 2.2 General bioinformatic data analyses

Data was imported and analyzed using the R statistical environment (v4.2) (19) using several packages obtained from the Bioconductor (v3.16) (20) repository. The analytical workflow was orchestrated by Snakemake (v7.14.0) (21). Visualizations were created using the *tidyverse* (v1.3.1) (22), *ggplot2* (v3.4.2) (23), *ggrastr* (v1.0.1), *ggrepel* (v0.9.3), *cowplot* (v1.1.1), *viridis* (v0.6.3) (24), *pheatmap* (v1.0.12).

### 2.3 Single-cell RNA-sequencing analysis

Samples were removed from the cryostat and thawed on ice. Thawed PBMCs were washed and then labelled using the BioLegend TotalSeq-B cell hashtag oligo (HTO) antibodies for multiplexing purposes per manufacturer’s protocol at 1 U per 1 million cells (25). An aliquot of the tagged PBMCs was assessed for viability using the Countess II FL Automated Cell Counter indicating that over 80% of the cells were viable. The resulting oligo-tagged cell suspensions were then mixed and distributed across 6 GEM-wells to be loaded onto the Chromium controller (10X Genomics) using 10X chemistry v3. Per well, 10,000 cells were loaded for a targeted recovery rate of up to 6,000 cells. Single-cell barcoded partitions were prepared using 10X chemistry v3 where after separate sequencing libraries were prepared for HTOs and the actual mRNA after size-selection. Libraries were sequenced on the Illumina HiSeq4000 in a 150 bp paired-ended fashion at the Core Facility Genomics, Amsterdam UMC. The mRNA libraries were sequenced on 150M reads per GEM-well, whereas the HTO libraries were sequenced to a depth of 50M reads per GEM-well.

Raw reads from both our own experiment were aligned and unique molecular identifier (UMI) count matrices were generated using Cellranger (v7.0.0) (10X Genomics). Subsequent import, sample-wise demultiplexing, processing, and analysis was done in Seurat (v4.3.0) (26). Cells that were identified as multiplets, based on the presence of an equal number of different HTOs, or that did obtain sufficient HTO signal were removed as they could not be assigned to a unique donor. Subsequent quality control included identifying dead cells based on mitochondrial read content (>75%) and a low number of unique genes, which were annotated accordingly (27). UMI counts were normalized using SCTransform (28) using default parameters. Cells were subsequently annotated by mapping our data onto a reference PBMC CITE-seq experiment of 162,000 annotated cells using a weighted nearest neighbor approach (29, 30). A subsequent manual curation using canonical markers confirmed the identity of the different cell types at cluster level. A dead and debris cluster was identified as cells with a low number of unique genes (<500 unique genes) and a high percentage mitochondrial reads (>80%), multiplet were identified on hashtags derived from multiple different donors (inter-donor multiplets), and/or a high number of unique genes (>2000 unique genes) in combination with multiple celltype-specific markers (mixed-cell multiplets). Proliferating cells were identified based on their high expression of proliferation marker *MKI67 (* 31). T cells were identified based on the expression of *CD3D*, *CD2*, *CD7*, and *IL7R*. Natural killer (NK) cells were identified based on the expression of *CD2*, *CD7*, *GNLY* and *NKG7*, while lacking *CD3D*, B-cells were identified based on the expression of *MS4A1* and *BANK1* positive. Monocytes were identified based on the expression of *CST3* and *CD14* or *FCGR3A*. Conventional dendritic cells (cDC) were identified on expression of *CD1C*, *CST3*, *FCER1A* and *HLA-DRA*. Smaller cell populations not belonging to the lineages were identified as well, namely the thrombocytes (*CST3* and *PPBP* positive), hematopoietic stem and progenitor cells (HSPCs; *CD34* positive), and erythroblasts (*HBA1*, *HBA2*, *HBQ1*, and *HBB* positive) (32).

The public single-cell RNA-sequencing data from Martin et al. (33) were obtained in the form of.fastq.gz files from the Sequence Read Archive (SRA). Preprocessing the data was analogous to our own single-cell RNA-sequencing analysis. Immune and epithelial cells were identified based on the expression of *PTPRC* and *EPCAM*, respectively. The cDCs were identified based on expression of *HLA-DRA* and *ITGAX*, whereas the plasmacytoid dendritic cells (pDCs) were identified based on the expression of *HLA-DRA*, *IL3RA* and *CLEC4C* (34).

### 2.4 Mass cytometry by time-of-flight

Concurrent with the single-cell RNA-sequencing analyses, mass cytometry by time of flight (CyTOF) was performed on a separate aliquot of the PBMC samples. Here, we measured the cell-surface expression of 37 proteins with a particular focus on the T cell lineage. An overview of all the antibodies used and their clones can be found in **Supplementary Table S1**. Cryopreserved PBMCs were thawed, washed with PBS, and resuspended in RPMI medium. Cellular viability was assessed through live/dead staining using 5μM Cisplatin in PBS at room temperature. Cisplatin signal was quenched by washing with Cell Staining Buffer (CSB; Fluidigm) after 5 minutes and washed away. As several targets in the panel are known to lose their binding specificity after PFA fixation, the corresponding antibodies were incubated in the presence of Human TruStain FcX™ Fc Receptor Blocking Solution (BioLegend) at room temperature for 30 minutes. After washing, cells were fixed with 1.6% PFA and labeled using the Cell-ID 20-Plex Pd Barcoding Kit (Fluidigm) for multiplexing purposes per manufacturer’s protocol. Pooled cells were then stained for remaining cell-surface targets. Antibody concentrations were optimized for staining 3M cells per 100 μL of CSB for 30 minutes at room temperature. For intracellular staining, cells were washed and incubated with antibodies for intracellular markers (CES1 and CTLA4). CES1 lacked a metal reporter but was the only rabbit anti-human antibody, so a goat anti-rabbit antibody coupled to ^175^Lu was used as a secondary staining for CES1. After washing with CSB, antibodies were again fixed with 1.6% PFA, washed and incubated overnight with ^191/193^Ir DNA intercalator (1:4000) diluted in Fix-and-Perm Buffer (Fluidigm). Cells were subsequently washed before data acquisition was performed on the CyTOF3-Helios (Fluidigm). After data acquisition, raw. FCS files were imported in R. Expression values were arcsinh-transformed with cofactor 5. Signal intensities and sample acquisition rates were reviewed for stability over time and events gated based on the condition that the flow was stable, excluding calibration beads, and within the 90th percentile of all Gaussian parameters. Resulting singlets were selected for CD45^+^ signal (**Supplementary Figure S1**). Cells were clustered in an unsupervised manner using the FlowSOM-package, where initial SOM-clustering was set to 300 clusters, using markers listed in **Supplementary Table S1**. The 300 clusters were subsequently manually metaclustered according to their phenotypic lineages, whereafter cells were annotated. UMAP dimensionality reduction was performed using the *uwot* (v0.1.14). A subsample of 16,000 cells were randomly selected without replacement for visualization purposes to approximately match the number of cells identified through scRNAseq, making the observations more comparable, the full dataset was used for quantitative analyses.

### 2.5 Flow cytometry of the plasmacytoid dendritic cells

In addition to using an aliquot of PBMCs of the same patients analyzed for scRNAseq and CyTOF, an additional two patients (1 responder and 1 non-responder) were included in the flow cytometry analyses. Upon thawing, PBMCs were washed in PBS and stained for a Live/Dead™ Fixable Near-IR Dead Cell Stain Kit (Invitrogen/Life Technologies, Amsterdam, Netherlands). Cells were subsequently stained for surface markers in FACS buffer (0.5% BSA, 0.01% NaN3 in PBS) and Human TruStain FcX™ Fc Receptor Blocking Solution (BioLegend) using the following antibodies: CD11c-PerCP Cy5.5 (clone: S-HCL-3, BioLegend), HLA-DR-Alexa Fluor 700 (clone: LN3, eBioscience), CD123-FITC (clone: 6H6, BioLegend), CD1C-PE-Cy7 (clone: L161, BioLegend), pan-lineage (CD3/CD19/CD20/CD56)-APC (clones: UCHT1;HIB19;2H7;5.1H11, BioLegend), CD14-BD Horizon V500 (clone: M5E2, Becton Dickinson) and CD16-PE (clone: 3G8, Becton Dickinson). Acquisition was performed on the BD LSR Fortessa™. Doublets were excluded and live single cells identified using the forward scatter height (FSC-H) versus the forward scatter area (FSC-A) and the side scatter height (SSC-H) versus side scatter area (SSC-A). Live cells were identified using the dead marker. Classical monocytes were defined as (T/B/NK) Lin^-^HLA−DR^+^CD14^++^CD16^-^, intermediate monocytes as Lin^-^HLA−DR^+^CD14^++^CD16^+^, and non-classical monocytes as Lin^-^HLA-DR^+^CD14^+^CD16^+^. Conventional dendritic cells (cDCs) were defined as (T/B/NK) Lin^-^HLA-DR^+^CD11c^+^CD1c^+^ and plasmacytoid DCs (pDCs) as Lin^-^HLA-DR^+^CD11c^-^CD123^+^. Fluorescence minus one (FMO) was used for gating and median fluorescence intensity was determined to quantify cell surface expression. An overview of all antibodies and their clones used can be found in **Supplementary Table S2**.

### 2.6 Flow cytometry of PBMCs from the EARNEST trial

The EARNEST trial is a completed randomized double-blind placebo-controlled trial where the efficacy of VDZ was assessed in UC patients with chronic pouchitis. Cryopreserved PBMCs were obtained from one VDZ- and one placebo-treated patient before and at week 14 of VDZ treatment and subjected to flow cytometric analyses for T-cells (CD3^+^) and integrin α_4_β_7_ through fluorescently-labeled VDZ.

### 2.7 RNA-sequencing of the classical monocytes

Akin to the flow cytometric analyses, PBMCs were washed in PBS and stained for a live/dead cell viability marker (LifeScience, Amsterdam, the Netherlands) alongside the antibodies mentioned above. Cell sorting was conducted on the SH800 Cell Sorter (Sony). Classical, intermediate, and non-classical monocytes were identified as (T/B/NK)Lin^-^HLA-DR^+^CD14^++^CD16^-^, HLA-DR^+^CD14^++^CD16^+^, and HLA-DR^+^CD14^+^CD16^+^, respectively. The classical monocytes were sorted out and were subsequently processed for RNA sequencing. Due to low input material, classical monocytes mRNA was converted into cDNA using the Ovation RNA-seq System V2 kit (NuGEN; Agilent, Santa Clara, United States), whereupon sequencing libraries were prepared using the Ovation Ultralow System V2 kit (NuGEN; Agilent, Santa Clara, United States) and thereafter sequenced in a 150 bp paired-ended fashion on the Illumina NovaSeq6000 to a depth of 40 million reads at the Amsterdam UMC Core Facility Genomics. Quality control of the raw reads was done using FastQC (v0.11.8) (35) and MultiQC (v1.0) (36). Raw reads were aligned to the human genome (GRCh38) using STAR (v2.7.0) and annotated using the Ensembl (v95) annotation (37). Post-alignment processing was performed through SAMtools (v1.9) (38), after which reads per gene were counted using the featureCounts function found in the Subread package (v1.6.3) (39, 40).

### 2.8 Statistics

Differential abundance analyses were conducted by comparing the proportions using a t-test as implemented in the *speckle* (v1.40.0) (40) and *limma* (v3.60.6) packages where we omitted cell types that were represented by 10 cells or less. Differential expression analyses were conducted using the “pseudobulk” approach (41) to account for cells coming from the same donor. The actual differential expression analyses were performed using the Wald test as implemented in the *DESeq2* (v1.36.0) (42) package. Differential abundance and expression analyses were corrected for sex and age using the following design matrix: ~ *Response* + *Sex* + *Age*. Subsequent gene set enrichment analyses were conducted using Wald statistic as input for *fgsea* (v1.22.0) (43) against the Kyoto Encyclopedia for Genes and Genomes (KEGG) gene sets (44). Sender/receiver analysis was performed by extracting the significantly differentially expressed genes (DEGs) in CD14+ monocytes belonging to the KEGG cytokine-cytokine receptor signaling pathway. These cytokine-cytokine receptor DEGs were then filtered for known receptors or ligands as documented in the ligand receptor resource provided by NicheNet (v1.1.1) (45), where after we filtered DEGs in all cell types for binding partners.

### 2.9 Study approval

All included patients provided informed consent and the sampling was in accordance with the institutional ethics committee (METC reference number: NL53989.018.15).

## 3 Results

### 3.1 Cohort assembly

**Figure 1:**
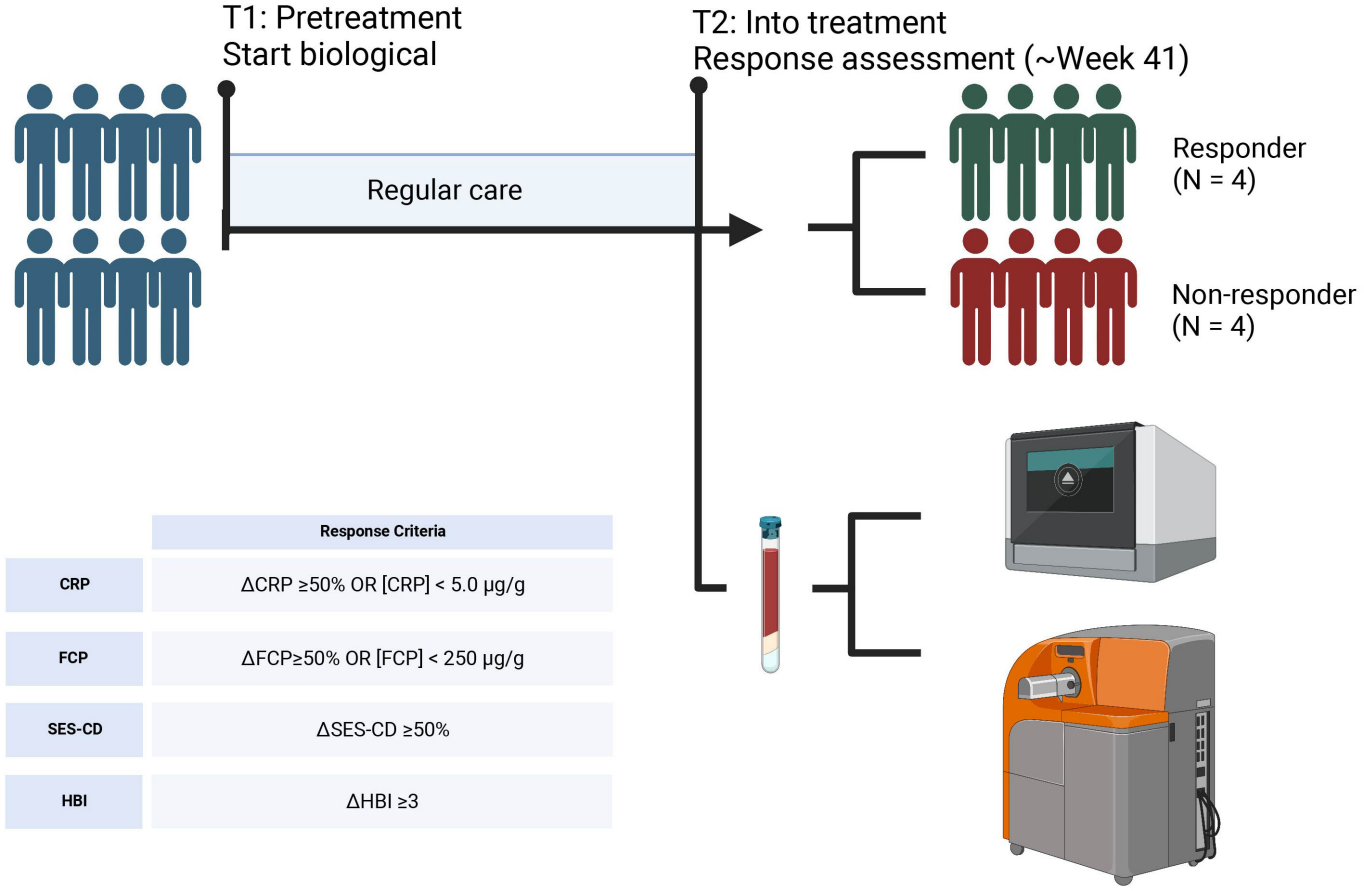
Sampling strategy. Peripheral blood samples were obtained from 4 CD patients that responded to VDZ and 4 CD patients that did not respond to VDZ. Response was defined based the Harvey Bradshaw Index (HBI), C-reactive protein (CRP), fecal calprotectin (FCP), and simple-endoscopic score CD (SES-CD). From peripheral blood, peripheral blood mononuclear cells (PBMCs) were isolated which were subsequently used for single-cell RNA-sequencing and mass cytometry by time of flight (CyTOF) using the Chromium controller (10X Genomics) and CyTOF3-Helios systems (Fluidigm), respectively. Created with BioRender.

Peripheral blood samples were obtained from a cohort of patients with CD on VDZ treatment at the AmsterdamUMC, location AMC as part of routine care. Response to treatment was defined as endoscopic- (≥50% drop in simple endoscopic score for Crohn’s Disease (SES-CD)) in combination with biochemical (≥50% reduction in C-reactive protein (CRP) and fecal calprotectin (FCP) or an absolute CRP<5.0 µg/g and FCP<250 µg/g) and/or clinical response (≥3 point drop in Harvey-Bradshaw Index (HBI)) compared to the start of treatment. All patients presented with measurable drug serum concentrations and no concomitant corticosteroid usage. For this study, we selected a cohort of 8 CD patients at a median of 41 weeks into VDZ treatment, which were classified as responder (N = 4) and non-responder (N = 4) (Methods, **Table 1**, **Figure 1**).

### 3.2 ITGA4 gene and protein expression detected on all PBMCs

**Figure 2:**
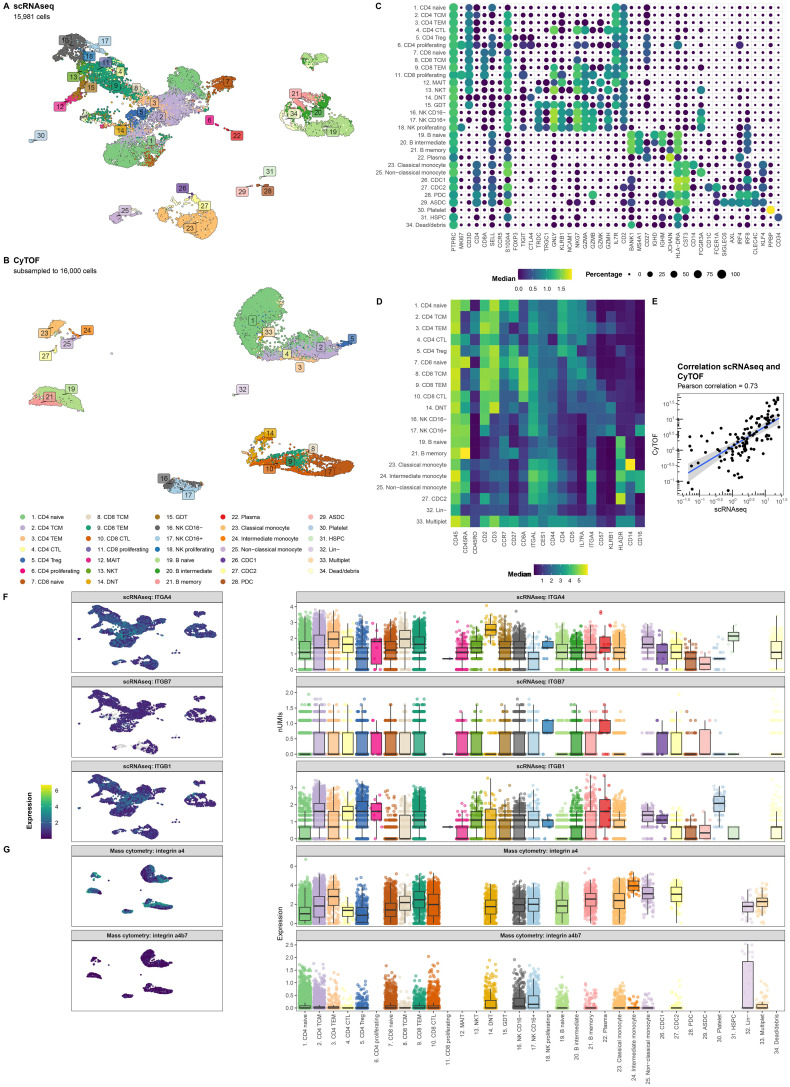
*ITGA4* is expressed by all cell types. Uniform manifold approximation and projection (UMAP) visualization of the PBMCs from CD patients on VDZ that respond (R; N = 4) and that do not respond (NR; N = 4) colored by the cellular identity as obtained through **(A)** single-cell RNA-sequencing (scRNAseq) and **(B)** mass cytometry by time of flight (CyTOF). Visualization of the marker expression used to annotate the PBMCs at the level of **(C)** gene expression through a dotplot where size and color intensity represent the percentage cells with measurable expression and the median expression, respectively, and **(D)** protein expression through a heatmap with the color representing the median expression. **(E)** Scatterplot representing the percentage cell types per sample relative to all PBMCs for scRNAseq on the X-axis and CyTOF on the Y-axis colored by lineage show general agreement between the scRNAseq and CyTOF experiment. UMAP (left) and boxplot (right) visualization of the gene expression for **(F)** *ITGA4*, *ITGB7*, *ITGB1* as well as **(G)** the protein expression for integrin α_4_ and the heterodimer integrin α_4_β_7_ per cell type. Colors in the UMAP visualization represent the level of expression per cell where grey represents no measurable expression.

Single-cell RNA-sequencing (scRNAseq) and mass cytometry by time of flight (CyTOF) provided transcriptional and proteomic profiles of 15,981 and 1,783,641 (subsampled to 16,000 for visualization purposes) cells, respectively. Cells were annotated to 31 known cell types (**Figures 2A, B**) using a combination of automatic and manual curation based on canonical markers (**Figures 2C, D**). We observed general agreement between the two experiments (r = 0.73; **Figure 2E**). Interrogating integrin α_4_ as well as its encoding gene *ITGA4* displayed measurable expression in all cell types (**Figures 2F, G**). By contrast, gene expression of *ITGB7* was notably muted, but still observable across all PBMCs with plasma cells presenting the highest expression. Indeed, interrogating the gene co-expression of *ITGA4* and *ITGB7* among all cell types indicates that most cells express *ITGA4* only but that most cells present some degree of co-expression of *ITGA4* and *ITGB7* (**Supplementary Figure S2**). As earlier observations indicated that heterodimer α_4_β_1_ may act as a redundant heterodimer by which effector T cells migrate to the gastrointestinal tract (46), we next interrogated *ITGB1* expression. We found that *ITGB1* expression was generally higher than *ITGB7* (**Figure 2F**). While *ITGB1* was most prominent among T cell subsets, all PBMCs presented reasonable expression of *ITGB1*. Indeed, a larger fraction of each cell type presented co-expression of *ITGA4* and *ITGB1* compared to *ITGA4* and *ITGB7* (**Supplementary Figure S3**). Finally, we tried to quantify the expression of heterodimer α_4_β_7_ through CyTOF by conjugating VDZ with a metal-tag. However, the median signal for all cell types was 0 (**Figure 2G**). Flow cytometry of stored PBMCs obtained from UC patients with chronic pouchitis from the EARNEST trial (47) using a fluorophore-conjugated VDZ conferred measurable signal only prior to the start of VDZ treatment, which disappeared during treatment, suggesting that VDZ treatment results in the loss of a conjugated VDZ signal (**Supplementary Figure S4**). Taken together, genes encoding integrin α_4,_ β_1_ and to a lesser extent β_7_ are expressed in many PBMC cell types and are hence not solely restricted to the T cells.

### 3.3 Circulating T cells from VDZ responders present pro-inflammatory phenotype

**Figure 3:**
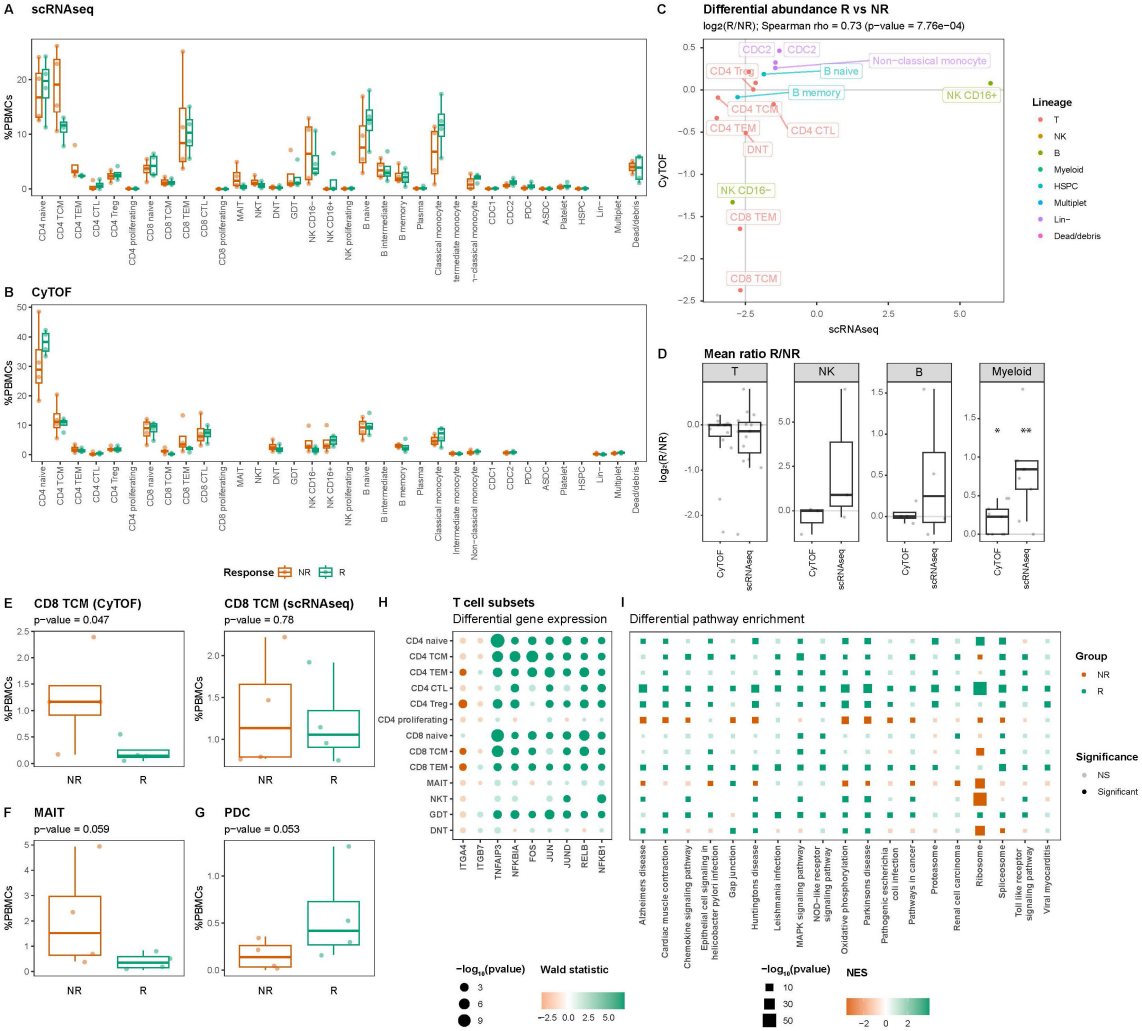
T cells present response-associated differences in abundance and expression. Boxplot visualizations of the cell type abundances relative to all measured PBMCs colored by response for **(A)** scRNAseq and **(B)** CyTOF. **(C)** Scatterplot comparing the differences in abundance based on scRNAseq on the X-axis and CyTOF on the Y-axis for cell types identified using both scRNAseq and CyTOF quantified using the Spearman correlation coefficient. Values represent log_2_-transformed responder:non-responder ratios per cell type and colors represent the parent lineages of each cell type. **(D)** Boxplot visualizations of the log_2_-transformed responder:non-responder ratios grouped by modality shows that myeloid cells are significantly more and less abundant amongst non-responders, respectively. Asterisks denote statistical significance using a one-sample t-test against 0. *p<0.05; **p<0.01. Boxplot visualizations of the abundance **(E)** CD8 T central memory (CD8 TCM) in the (left) CyTOF experiment and (right) scRNAseq experiment, **(F)** mucosal associated invariant T (MAIT) cells, and **(G)** plasmacytoid dendritic cells (pDC) relative to all PBMCs. *P*-values were calculated using the t-test implementation in speckle::propeller. Dot- and square plot visualization representing **(H)** differential gene expression and **(I)** pathway enrichment analyses, respectively, showing lower expression of pro-inflammatory genes in non-responder-derived T cells relative to the responder counterparts. Size represents statistical significance, transparency the significance threshold, and color the effect size in Wald statistic (gene expression) or normalized enrichment score (NES; pathway enrichment) indicating whether the gene or pathway is upregulated in either responders (green) or non-responders (orange).

Differential abundance analysis of both the scRNAseq and CyTOF data indicated reasonably concordant differences between responders and non-responders for cell types measured using both modalities (Spearman rho = 0.73; *p*-value = 7.7E-04) (**Figures 3A-C**, **Supplementary Table S3**). Overall, non-responders presented with a significantly lower relative abundance of the myeloid cells, which was visible using both scRNAseq (nominal *p*-value = 2.6E-03) and CyTOF (nominal *p*-value = 0.02) (**Figure 3D**). At a more granular level, a nominally significantly higher abundance of CD8 T central memory (CD8 TCM) was observed through CyTOF (nominal *p*-value = 0.047), which we could reproduce in direction, but not in significance, through scRNAseq (nominal *p*-value = 0.78) (**Figure 3E**). At scRNAseq level, a nominally significantly higher abundance was observed for the mucosal associated invariant T cells (MAIT; nominal *p*-value = 0.059) (**Figure 3F**) and a lower abundance of plasmacytoid dendritic cells (pDCs; nominal *p*-value = 0.053) (**Figure 3G**), respectively, which we were unable to reproduce using CyTOF as no markers were included for either MAIT or pDCs. As VDZ reportedly binds T cells in particular (7), we investigated whether their transcriptome presented response-associated differences (**Supplementary Table S4**). We specifically interrogated *ITGA4* and *ITGB7* expression but found no differences in expression for *ITGB7*. By contrast, *ITGA4* was found to be significantly higher in non-responders when looking at CD4 TEM, CD4 Treg, CD8 TCM, and CD8 TEM. A more comprehensive differential expression analysis indicated that T cell subsets derived from non-responders presented lower expression of pro-inflammatory genes, such as *FOS*, *JUN*, *RELB*, and *NFKB1* (**Figure 3H**). Through gene set enrichment analyses, we indeed found that most T cell subsets from responders expressed lower levels of genes associated with Toll-like receptor (TLR), NOD-like receptor (NLR), and mitogen-activated protein kinases (MAPK) signaling compared to their responder counterparts (**Figure 3I**). Taken together, it seems that responder-derived T-cells present a more pro-inflammatory phenotype compared to their non-responder counterparts.

### 3.4 VDZ non-responders present higher relative abundances of circulating plasmacytoid dendritic cells

**Figure 4:**
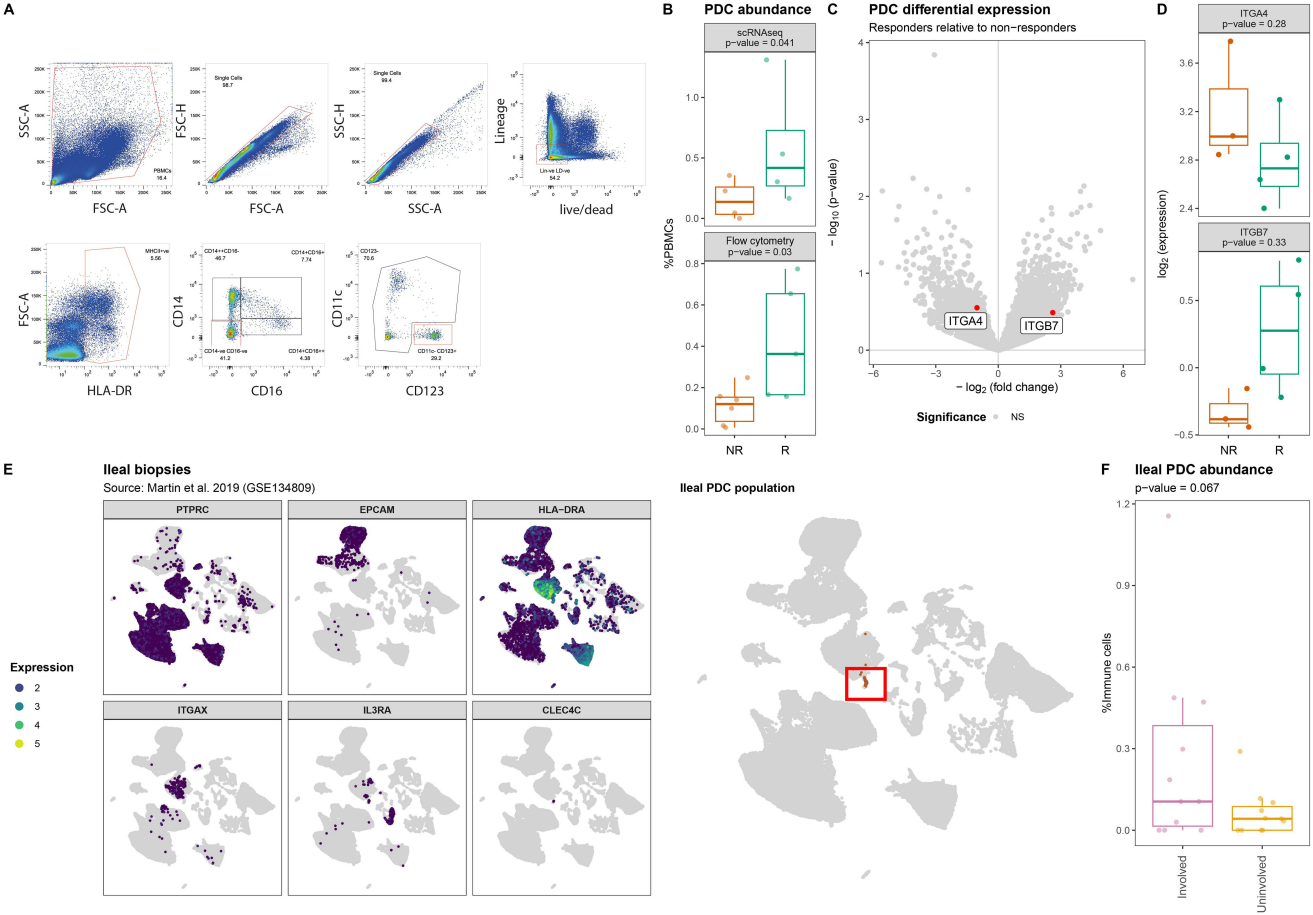
Lower abundance of plasmacytoid dendritic cells in PBMCs of non-responding patients. **(A)** Flow cytometry strategy used to identify and quantify the HLA-DR^+^CD14^-^CD16^-^CD11c^-^CD123^+^ pDCs where red boxes indicate selected events. **(B)** Boxplot visualizations of the pDC abundances relative to all measured PBMCs annotated with the *p*-value obtained through t-test. **(C)** Volcanoplot comparing pDCs from responders with non-responders where the X-axis represents the log_2_(fold-change) and the Y-axis the –log_10_(*p*-value). Highlighted in red are *ITGA4* and *ITGB7*. **(D)** Boxplot visualizations of the *ITGA4* and *ITGB7* expression in pDCs showing visible but no statistical significant differences between responders and non-responders. *P*-values were calculating using the t-test implementation in speckle::propeller. **(E)** UMAP visualization of GSE134809 showing (left) the identification strategy of the PTPRC[CD45]^+^EPCAM^-^HLA-DRA^+^ITGAX[CD11c]^-^IL3RA[CD123]^+^CLEC4C[BDCA2]^+^ pDCs, (right) as highlighted by the red box. **(F)** Boxplot visualization of the ileal pDC abundance relative to all immune cells colored by whether they originate from lesions (involved) or outside a lesion (uninvolved) shows that lesional pDCs are more abundant than non-lesional pDCs.

As our CyTOF panel did not include markers for pDCs, we conducted flow cytometry analyses where we identified the (T/B/NK)Lin^-^HLA-DR^+^CD11c^-^CD123^+^ pDC fraction (**Figure 4A**). Indeed, we observed a significantly lower proportion of circulatory pDCs among responders relative to the non-responders (*p*-value = 0.03) (**Figure 4B**). However, interrogating the transcriptome of the pDCs did not identify statistically significant response-associated differences between responders and non-responders after correcting for multiple testing (**Figure 4C**, **Supplementary Table S5**). Notably, the expression of *ITGA4* and *ITGB7* specifically indicated a lower expression of *ITGB7* amongst non-responders albeit statistically non-significant (nominal *p*-value = 0.37) (**Figure 4D**, **Supplementary Table S5**). We hypothesized that the diminished proportion of circulatory pDCs among non-responders was due to their recruitment into the gastrointestinal tract thereby removing them from circulation. To corroborate our hypothesis, we interrogated the publicly available single-cell transcriptomic data from CD patients’ intestinal biopsies extracted from ileal lesions (involved) and adjacent non-lesional (uninvolved) tissue as published by Martin et al. (33). Upon identifying the pDC fraction (**Figure 4E**), we found that the pDC proportion relative to the total immune fraction was suggestively higher in lesional compared to non-lesional areas (nominal *p*-value = 0.067) (**Figure 4F**) indicating that the relative abundance of pDCs is higher under inflammatory conditions, thereby supporting our hypothesis.

### 3.5 Classical monocytes from VDZ non-responders present an altered transcriptome

**Figure 5:**
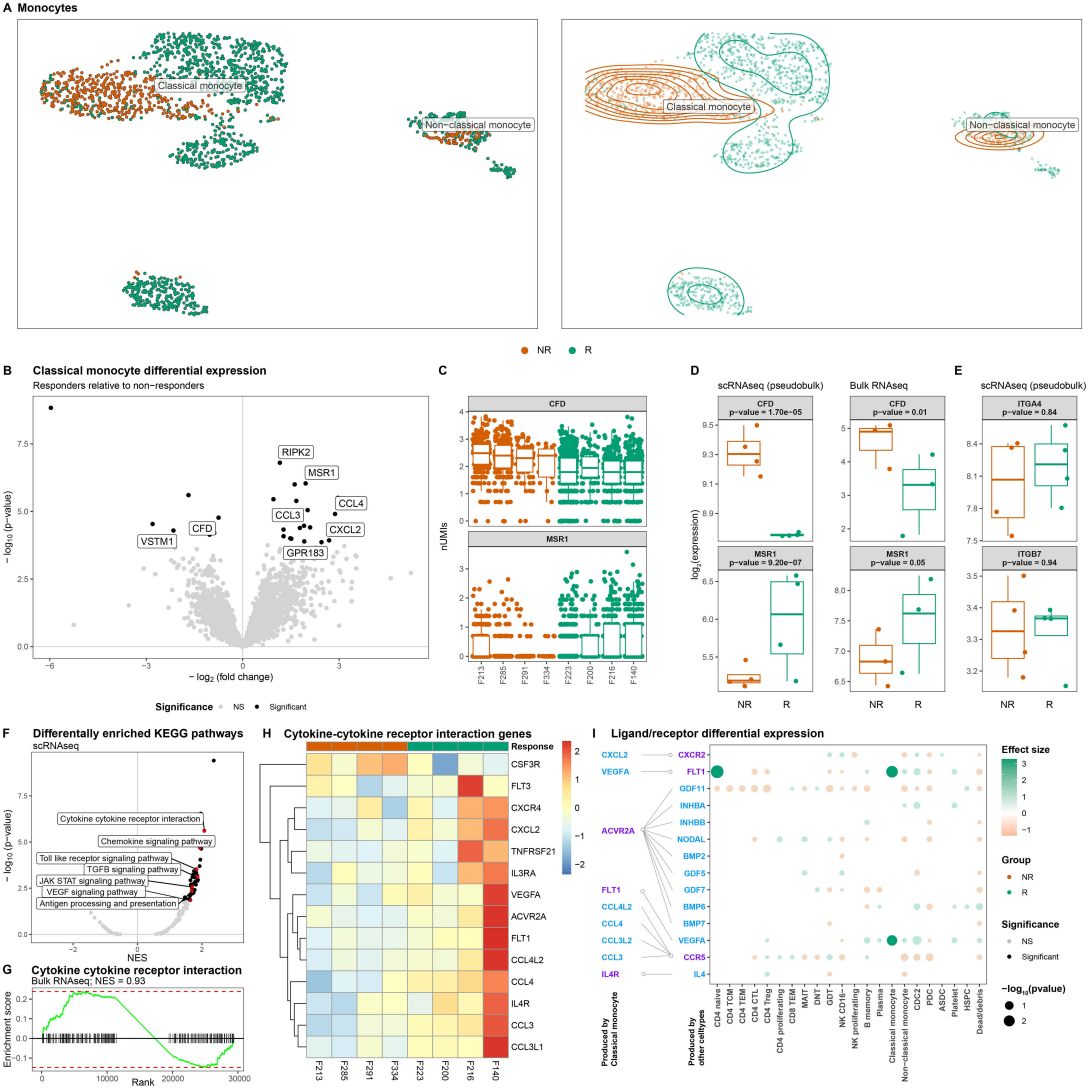
Classical monocytes from non-responding patients present lower expression of cytokine-cytokine signaling. **(A)** UMAP visualization of the monocytes colored by colored by response as dots (left) and a density plot (right) shows distinct clustering by response for the classical monocytes in particular. **(B)** Volcanoplot comparing classical monocytes from responders with non-responders where the X-axis represents the log_2_(fold change) and the Y-axis the –log_10_(*p*-value). Statistically significant differences (*p*-value_BH-adjusted_<0.05) are depicted in black. **(C)** Boxplot visualizations of *CFD* and *MSR1* expression in classical monocytes colored by response and grouped by patient where each dot represents an individual cell. Boxplot visualizations of **(D)** *CFD* and *MSR1*, and **(E)** *ITGA4* and *ITGB7* where the Y-axis represents gene log-transformed (left) normalized pseudobulk expression from our scRNAseq experiment and (right) normalized expression obtained through bulk RNAseq analysis on classical monocytes colored and grouped by response. *P*-values were obtained through Wald test as implemented in DESeq2. **(F)** Volcanoplot of the gene set enrichment analysis obtained from scRNAseq comparing classical monocytes from responders with non-responders where the X-axis represents the normalized enrichment score (NES) and the Y-axis the –log_10_(*p*-value). Statistically significant differences (*p*-value_BH-adjusted_<0.05) are depicted in black with pathways of interest highlighted in red. **(G)** Barcode plot visualizing the enrichment of cytokine-cytokine receptor interaction pathway as obtained through bulk RNAseq of the classical monocytes. **(H)** Heatmap visualization genes belonging to the cytokine-cytokine receptor interaction pathway. Values represent the pseudobulk expression per sample, where color is proportional to the level of expression. **(I)** Dotplot visualization of a receptor/ligand interaction analysis of the differentially expressed cytokines representing ligands and receptors are colored in blue and purple, respectively. Depicted in the dotplot are the binding partners of the cytokines found to be differentially expressed by the classical monocytes for each cell type found in PBMCs. Size of the dots represents statistical significance, transparency the significance threshold, and color whether the gene is upregulated in either responders (green) or non-responders (orange). We find that VEGFA receptor FLT1 is significantly higher expressed among the CD4 naïve and classical monocytes of responders.

UMAP visualization of the monocytes indicated response-associated clustering (**Figure 5A**), which was most visible for the classical monocytes, suggesting transcriptome-wide differences. Differential expression analysis of the classical monocytes identified 30 statistically significant differentially expressed genes (DEGs) (**Figure 5B**, **Supplementary Table S6**). Notably, responders presented higher expression of several monocyte/macrophage-function related genes including genes encoding cytokines (CXCL2 (48, 49), CCL3 (50–52), CCL4 (53, 54)), mediators of host defense signaling (RIPK2 (55)), and macrophage scavenging receptor (MSR1 (56)), typically observed in M2-like macrophages. By contrast, expression of genes encoding complement factor D (*CFD*) and negative regulator of NFκB signaling pathway (*VSTM1*) (57) was higher among non-responders (**Figure 5C**). We were able to confirm differential expression for *CFD* and *MSR1* through bulk RNA-sequencing on sorted classical monocytes (**Figure 5D**, **Supplementary Table S7**). Specifically interrogating *ITGA4* and *ITGB7* indicated neither significant nor visible differences in the gene expression thereof (**Figure 5E**). Gene set enrichment analysis of the pseudobulk scRNAseq data against the KEGG database identified general lower expression of cytokine-cytokine receptor signaling pathways among non-responders, which we specified to the TLR-, TGFβ-, JAK STAT-, and VEGF-signaling pathways (**Figures 5F-H**, **Supplementary Table S8**). Our bulk RNA-sequencing analysis on the classical monocytes validated the lower expression of cytokine-cytokine receptor signaling pathway among non-responders (**Figure 5G**). We were therefore interested in identifying whether the classical monocytes were tentatively communicating with other cell types. To this end, we conducted sender/receiver analyses of the differentially expressed cytokines produced by the classical monocytes (**Figure 5I**). Among classical monocytes derived from responders, we observed a significantly higher expression of vascular endothelial growth factor (VEGF). Notably, VEGF receptor 1-encoding *FLT1* was found to be higher in both the CD4T naïve as well as the classical monocytes among responders (**Figure 5H**), corroborating the enriched the VEGF signaling pathway (**Figure 5F**).

## 4 Discussion

We demonstrate that VDZ-treated CD patients differ in cellular composition and intrinsic cellular behavior when comparing responders with non-responders. We confirmed that all PBMCs generally express both gene and protein of integrin α4, but note a muted in the gene expression of *ITGB7*. By contrast, *ITGB1* appears to be reasonably co-expressed alongside *ITGA4* across all PBMCs, which could be the result of a bypass mechanism by which leucocytes could migrate to the gastrointestinal tract in the presence of VDZ (46). We failed to present the expression of heterodimer integrin α4β7 through mass cytometry, which we hypothesize is due to the presence of regular VDZ during treatment outcompeting the metal-tagged VDZ, evidenced by earlier flow cytometric analyses of fluorophore-tagged VDZ prior to and during VDZ treatment.

Interrogating the T cell compartment suggested a higher abundance of MAIT and CD8 TCM among non-responders. Notably, most T cells from responders appeared to be transcriptionally primed for the TLR, NLR, and MAPK signaling pathways, which are typically described as pro-inflammatory. We are unsure why responder-associated T cells present a more pro-inflammatory phenotype compared to their non-responder counterparts, but hypothesize that VDZ treatment of non-responding patients does not prevent their recruitment out of circulation.

We next observed that the circulatory pDCs were significantly less abundant amongst non-responders, which we were able to correlate an observable higher abundance in lesional compared non-lesional ileal tissue obtained from Martin et al. (33). The pDC population represents a unique cell type whose ontogeny and lineage affiliation remain under debate due to its ability to derive from both myeloid and lymphoid lineages (34, 58–61). Previously, pDCs were called “natural interferon producing cell” as they can produce large amounts of type I interferons (IFN), which typically occurs in response to viruses. This in turn activates NK and B cells (62–65), thereby bridging the innate and adaptive immune system. Remarkably, pDCs constitute only 0.4% of all measured cells when looking at all measured PBMCs in our scRNAseq experiment and only 0.12% of the immune compartment when interrogating ileal tissue (33). Despite the rarity of the pDC population, they have been implicated in multiple immune-mediated inflammatory disorders (IMIDs) (61) evidenced by an ongoing phase II clinical trial testing the efficacy of litifilimab, a monoclonal antibody against pDC-specific blood dendritic cell antigen 2 (BDCA2), in systemic (66) and cutaneous (67) lupus erythematosus to dampen type I IFN production (68, 69). Despite the established role of pDCs in other IMIDs, their association with CD or IBD as a whole is less well documented. Previous studies have indicated that the circulatory pDC population is significantly decreased in IBD patients with active disease (70), with subsequent research by the same authors showing increased infiltration into the colonic mucosa and mesenteric lymph node (MLN) (71). This largely corroborates our own observations, as samples were obtained during treatment and the difference between responders and non-responders is by definition a difference in inflammation. However, controversy exists on what role pDCs play in the pathogenesis of IBD as experiments have yielded conflicting results. It has been reported that pDCs can aggravate (72), protect (73, 74), or are dispensable in the development of experimental IBD (75). Accordingly, it remains unclear how pDCs might play a role in responsiveness towards VDZ.

Significant differences in expression were observed for the circulating classical monocytes, which presented a enrichment of the TGFβ signaling and VEGF receptor pathway amongst responders, suggesting response-associated differences in angiogenic behavior. By contrast, classical monocytes from non-responders appeared to present higher expression of *CFD*, a gene involved in the alternative complement pathway. Complement factor D cleaves factor B forming Bb, thereby activating the complement cascade (76). The alternative complement pathway is an important component of the innate immune response where it is typically used as first line defense against microbes. Our results would imply that the classical monocytes from non-responders are primed at activating the alternative complement pathway. Such monocytes could potentially be recruited into the intestinal compartment, where they would differentiate into macrophages. Intestinal inflammatory macrophages are one of the few macrophages that are purported to be supplemented by the circulating monocyte population during inflammatory episodes (77–79). We show that monocytes indeed express genes encoding components that form integrin α_4_β_7_, corroborating earlier observations by Schleier et al. who showed that monocytes present functional integrin α_4_β_7_ on their surface, with VDZ abrogating their interactions with MAdCAM-1 *in vitro* (12). While our observations do not indicate any difference in expression of either *ITGA4* or *ITGB7*, we note that the classical monocytes, alongside all other myeloid cells, were less abundant among non-responders, which we hypothesize is due to their recruitment out of circulation and into the intestinal compartment.

While we provide novel insights into the diagnostic capabilities of single-cell transcriptomics for elucidating response to VDZ, we acknowledge that the current exploratory study is limited by its size as well as the observed patient heterogeneity evidenced by differences between responders and non-responders in terms of disease location, treatment duration and history, and smoking behavior (**Table 1**). Future studies may want to investigate whether disease location, treatment history, and smoking behavior significantly affect the single cell transcriptome of PBMCs. Importantly, we note that response in our patient cohort was primarily based on clinical and biochemical endpoints, as patients from both responders and non-responders presented reasonable improvements in their endoscopic response, which represents the best metric for predicting mucosal healing (80). Future studies would therefore need to be conducted in a larger, independent, homogeneous patient cohort consisting of proper endoscopic responders and non-responders to fully understand its potential utility as biomarker for treatment-induced mucosal healing. Another limitation of the current study is that the current observations remain largely associative and provide limited mechanistic insights into response to medication, which manifests primarily within the gastrointestinal tract. For biomarker purposes, peripheral blood serves as an easily accessible tissue over the more invasive endoscopies. However, intestinal biopsies would have likely provided more biological insights. Finally, patient-samples were taken during treatment at different timepoints after start of treatment and therefore do not hold prognostic value in predicting response to therapy. To properly disentangle inflammation from VDZ-treatment, samples would need to be included prior to the start of treatment. It is imperative to compare our observations with CD patient cohorts treated with other inflammatory-reducing medications, such as anti-TNF, to understand which observations are VDZ-specific and which observations are inflammation-associated. Such an approach would not only allow for the identification of prognostic biomarkers for VDZ response, but also provide potential targets that might be involved in the manifestation of drug non-response.

In summary, our exploratory study indicate that response to VDZ during treatment manifests itself in various circulating cell types, presenting differences in both abundance as well as expression. Further confirmatory studies are necessary to validate and further understand the full potential of the observed differences.

## Data Availability

The processed counts for the single-cell RNA-sequencing performed on PBMCs and the bulk RNA-sequencing performed on CD14+ monocytes are publicly available. This data can be found here: 10.5281/zenodo.16786994.

## References

[1] Sandborn WJ Feagan BG Rutgeerts P Hanauer S Colombel J-F Sands BE. Vedolizumab as induction and maintenance therapy for crohn’s disease. The New England Journal of Medicine (2013) 369:711–21. doi: 10.1056/NEJMOA1215739, PMID: 23964933

[2] Meenan J Spaans J Grool TA Pals ST Tytgat GNJ Van Deventer SJH. Altered expression of α4β7, a gut homing integrin, by circulating and mucosal T cells in colonic mucosal inflammation. Gut. (1997) 40:241–6. doi: 10.1136/gut.40.2.241, PMID: 9071939 PMC1027056

[3] Gorfu G Rivera-Nieves J Ley K. Role of 7 integrins in intestinal lymphocyte homing and retention. Journal: Current Molecular Medicine (2009) 9:836–50. doi: 10.2174/156652409789105525, PMID: 19860663 PMC2770881

[4] Yang Y Cardarelli PM Lehnert K Rowland S Krissansen GW. LPAM-1 (integrin α4β7)-ligand binding: Overlapping binding sites recognizing VCAM-1, MAdCAM-1 and CS-1 are blocked by fibrinogen, a fibronectin-like polymer and RGD-like cyclic peptides. Eur J Immunol. (1998) 28:995–1005. doi: 10.1002/(SICI)1521-4141(199803)28:03<995::AID-IMMU995>3.0.CO;2-D, PMID: 9541595

[5] Berlin C Bargatze RF Campbell JJ Von Andrian UH Szabo MC Hasslen SR. a4 integrins mediate lymphocyte attachment and rolling under physiologic flow. Journal Cell (1995) 80:413–22. doi: 10.1016/0092-8674(95)90491-3, PMID: 7532110

[6] Hynes RO. Integrins: bidirectional, allosteric signaling machines. Cell. (2002) 110:673–87. doi: 10.1016/S0092-8674(02)00971-6, PMID: 12297042

[7] Soler D Chapman T Yang LL Wyant T Egan R Fedyk ER. The binding specificity and selective antagonism of vedolizumab, an anti-α4β7 integrin therapeutic antibody in development for inflammatory bowel diseases. J Pharmacol Exp Ther. (2009) 330:864–75. doi: 10.1124/jpet.109.153973, PMID: 19509315

[8] Arijs I De Hertogh G Lemmens B Van Lommel L de Bruyn M Vanhove W. Effect of vedolizumab (anti-α4β7-integrin) therapy on histological healing and mucosal gene expression in patients with UC. Gut. (2018) 67:43–52. doi: 10.1136/gutjnl-2016-312293, PMID: 27802155

[9] Lord JD Long SA Shows DM Thorpe J Schwedhelm K Chen J. Circulating integrin alpha4/beta7+ lymphocytes targeted by vedolizumab have a pro-inflammatory phenotype. Clin Immunol. (2018) 193:24–32. doi: 10.1016/j.clim.2018.05.006, PMID: 29842945 PMC6257989

[10] Habtezion A Nguyen LP Hadeiba H Butcher EC. Leukocyte trafficking to the small intestine and colon. Gastroenterology. (2016) 150:340–54. doi: 10.1053/j.gastro.2015.10.046, PMID: 26551552 PMC4758453

[11] Zeissig S Rosati E Dowds CM Aden K Bethge J Schulte B. Vedolizumab is associated with changes in innate rather than adaptive immunity in patients with/ inflammatory bowel disease. Gut. (2019) 68:25–39. doi: 10.1136/gutjnl-2018-316023, PMID: 29730603

[12] Schleier L Wiendl M Heidbreder K Binder MT Atreya R Rath T. Non-classical monocyte homing to the gut via α4β7 integrin mediates macrophage-dependent intestinal wound healing. Gut. (2020) 69:252–63. doi: 10.1136/gutjnl-2018-316772, PMID: 31092589

[13] Canales-Herrerias P Uzzan M Seki A Czepielewski RS Verstockt B Livanos A. Gut-associated lymphoid tissue attrition associates with response to anti-α4β7 therapy in ulcerative colitis. bioRxiv. (2023), 524731. doi: 10.1101/2023.01.19.524731, PMID: 38640252 PMC11140591

[14] Dulai PS Singh S Jiang X Peerani F Narula N Chaudrey K. The real-world effectiveness and safety of vedolizumab for moderate-severe Crohn’s disease: Results from the US VICTORY consortium. Am J Gastroenterol. (2016) 8:1147–55. doi: 10.1038/ajg.2016.236, PMID: 27296941

[15] Ungar B Kopylov U Yavzori M Fudim E Picard O Lahat A. Association of vedolizumab level, anti-drug antibodies, and α4β7 occupancy with response in patients with inflammatory bowel diseases. Clin Gastroenterol Hepatol. (2018) 16:697–705.e7. doi: 10.1016/J.CGH.2017.11.050, PMID: 29223444

[16] Perry C Fischer K Elmoursi A Kern C Currier A Kudaravalli P. Vedolizumab dose escalation improves therapeutic response in a subset of patients with ulcerative colitis. Dig Dis Sci. (2021) 66:2051–8. doi: 10.1007/S10620-020-06486-X/METRICS, PMID: 32710192

[17] Peyrin-Biroulet L Danese S Argollo M Pouillon L Peppas S Gonzalez-Lorenzo M. Loss of Response to Vedolizumab and Ability of Dose Intensification to Restore Response in Patients With Crohn’s Disease or Ulcerative Colitis: A Systematic Review and Meta-analysis (2019). Available online at: https://www.sciencedirect.com/science/article/pii/S1542356518306360?via%3Dihub (Accessed October 7, 2019). 10.1016/j.cgh.2018.06.026 29935327

[18] Joustra V Li Yim AYF Hageman I Levin E Noble A Chapman T. Highly stable epigenome-wide peripheral blood DNA methylation signatures accurately predict endoscopic response to adalimumab, vedolizumab and ustekinumab in Crohn’s disease patients: The EPIC-CD study. J Crohn’s Colitis. (2023), i6–8. doi: 10.1093/ecco-jcc/jjac190.0003

[19] R Development Core Team. R: A language and environment for statistical computing. Vienna, Austria: R Foundation for Statistical Computing (2008). Available online at: http://www.r-project.org/ (Accessed September 3, 2020).

[20] Gentleman RC Carey VJ Bates DM Bolstad B Dettling M Dudoit S. Bioconductor: open software development for computational biology and bioinformatics. Genome Biol. (2004) 5:R80. doi: 10.1186/gb-2004-5-10-r80, PMID: 15461798 PMC545600

[21] Köster J Mölder F Jablonski KP Letcher B Hall MB Tomkins-Tinch CH. Sustainable data analysis with Snakemake. F1000Research. (2021) 10:33. doi: 10.12688/f1000research.29032.2, PMID: 34035898 PMC8114187

[22] Wickham H Averick M Bryan J Chang W McGowan LD François R. Welcome to the tidyverse. J Open Source Softw. (2019) 4:1686. doi: 10.21105/joss.01686

[23] Wickham H. ggplot2: Elegant Graphics for Data Analysis. New York, NY: Springer-Verlag New York (2009). Available online at: http://ggplot2.org (Accessed September 3, 2020).

[24] Garnier S Ross N Rudis b Filipovic-Pierucci A Galili T timelyportfolio. viridis(Lite) - Colorblind-Friendly Color Maps for R. (2023). doi: 10.5281/zenodo.4679424. Rudis b, Filipovic-Pierucci A, Galili T, timelyportfolio,

[25] Stoeckius M Zheng S Houck-Loomis B Hao S Yeung BZ Mauck WM. Cell Hashing with barcoded antibodies enables multiplexing and doublet detection for single cell genomics. Genome Biol. (2018) 19:224. doi: 10.1186/s13059-018-1603-1, PMID: 30567574 PMC6300015

[26] Butler A Hoffman P Smibert P Papalexi E Satija R. Integrating single-cell transcriptomic data across different conditions, technologies, and species. Nat Biotechnol. (2018) 36:411–20. doi: 10.1038/nbt.4096, PMID: 29608179 PMC6700744

[27] Luecken MD Theis FJ. Current best practices in single-cell RNA-seq analysis: a tutorial. Mol Syst Biol. (2019) 15:e8746. doi: 10.15252/msb.20188746, PMID: 31217225 PMC6582955

[28] Hafemeister C Satija R. Normalization and variance stabilization of single-cell RNA-seq data using regularized negative binomial regression. Genome Biol. (2019) 20:296. doi: 10.1101/576827, PMID: 31870423 PMC6927181

[29] Stuart T Butler A Hoffman P Hafemeister C Papalexi E Mauck 3WM. Comprehensive integration of single-cell data. Cell. (2019) 177:1888–1902.e21. doi: 10.1016/j.cell.2019.05.031, PMID: 31178118 PMC6687398

[30] Hao Y Hao S Andersen-Nissen E Mauck WM Zheng S Butler A. Integrated analysis of multimodal single-cell data. Cell. (2021) 184:3573–3587.e29. doi: 10.1016/J.CELL.2021.04.048, PMID: 34062119 PMC8238499

[31] Tirosh I Izar B Prakadan SM Wadsworth MH Treacy D Trombetta JJ. Dissecting the multicellular ecosystem of metastatic melanoma by single-cell RNA-seq. Science. (2016) 352:189–96. doi: 10.1126/SCIENCE.AAD0501/SUPPL_FILE/TIROSH.SM.PDF, PMID: 27124452 PMC4944528

[32] Franzén O Gan LM Björkegren JLM. PanglaoDB: A web server for exploration of mouse and human single-cell RNA sequencing data. Database. (2019) 2019. doi: 10.1093/database/baz046, PMID: 30951143 PMC6450036

[33] Martin JC Chang C Boschetti G Ungaro R Giri M Grout JA. Single-cell analysis of crohn’s disease lesions identifies a pathogenic cellular module associated with resistance to anti-TNF therapy. Cell. (2019) 178:1493–1508.e20. doi: 10.1016/j.cell.2019.08.008, PMID: 31474370 PMC7060942

[34] Reizis B. Plasmacytoid dendritic cells: development, regulation, and function. Immunity. (2019) 50:37–50. doi: 10.1016/J.IMMUNI.2018.12.027, PMID: 30650380 PMC6342491

[35] Andrews S. FastQC: a quality control tool for high throughput sequence data . Available online at: http://www.bioinformatics.babraham.ac.uk/projects/fastqc (Accessed September 3, 2020).

[36] Ewels P Magnusson M Lundin S Kaller M. MultiQC: summarize analysis results for multiple tools and samples in a single report. Bioinformatics. (2016) 32:3047–8. doi: 10.1093/bioinformatics/btw354, PMID: 27312411 PMC5039924

[37] Cunningham F Allen JE Allen J Alvarez-Jarreta J Amode MR Armean IM. Ensembl 2022. Nucleic Acids Res. (2022) 50:D988–95. doi: 10.1093/NAR/GKAB1049, PMID: 34791404 PMC8728283

[38] Li H Handsaker B Wysoker A Fennell T Ruan J Homer N. The sequence alignment/map format and SAMtools. Bioinformatics. (2009) 25:2078–9. doi: 10.1093/bioinformatics/btp352, PMID: 19505943 PMC2723002

[39] Liao Y Smyth GK Shi W. The Subread aligner: fast, accurate and scalable read mapping by seed-and-vote. Nucleic Acids Res. (2013) 41:e108. doi: 10.1093/nar/gkt214, PMID: 23558742 PMC3664803

[40] Phipson B Sim CB Porrello ER Hewitt AW Powell J Oshlack A. propeller: testing for differences in cell type proportions in single cell data. Bioinformatics. (2022) 38:4720–6. doi: 10.1093/BIOINFORMATICS/BTAC582, PMID: 36005887 PMC9563678

[41] Squair JW Gautier M Kathe C Anderson MA James ND Hutson TH. Confronting false discoveries in single-cell differential expression. Nat Commun. (2021) 12:1–15. doi: 10.1038/s41467-021-25960-2, PMID: 34584091 PMC8479118

[42] Love MI Huber W Anders S. Moderated estimation of fold change and dispersion for RNA-seq data with DESeq2. Genome Biol. (2014) 15:550. doi: 10.1186/s13059-014-0550-8, PMID: 25516281 PMC4302049

[43] Sergushichev AA. An algorithm for fast preranked gene set enrichment analysis using cumulative statistic calculation. bioRxiv. (2016), 060012. doi: 10.1101/060012

[44] Kanehisa M Sato Y Kawashima M Furumichi M Tanabe M. KEGG as a reference resource for gene and protein annotation. Nucleic Acids Res. (2016) 44:D457–62. doi: 10.1093/nar/gkv1070, PMID: 26476454 PMC4702792

[45] Browaeys R Saelens W Saeys Y. NicheNet: modeling intercellular communication by linking ligands to target genes. Nat Methods. (2019) 17:159–62. doi: 10.1038/s41592-019-0667-5, PMID: 31819264

[46] Zundler S Fischer A Schillinger D Binder MT Atreya R Rath T. The α4β1 homing pathway is essential for ileal homing of crohn’s disease effector T cells *in vivo* . Inflammation Bowel Dis. (2017) 23:379–91. doi: 10.1097/MIB.0000000000001029, PMID: 28221249

[47] Travis S Silverberg MS Danese S Gionchetti P Löwenberg M Jairath V. Vedolizumab for the treatment of chronic pouchitis. New Engl J Med. (2023) 388:1191–200. doi: 10.1056/NEJMOA2208450/SUPPL_FILE/NEJMOA2208450_DATA-SHARING.PDF 36988594

[48] Pelus LM Fukuda S. Peripheral blood stem cell mobilization: The CXCR2 ligand GROβ rapidly mobilizes hematopoietic stem cells with enhanced engraftment properties. Exp Hematol. (2006) 34:1010–20. doi: 10.1016/J.EXPHEM.2006.04.004, PMID: 16863907

[49] Wolpe SD Sherry B Juers D Davatelis G Yurt RW Cerami A. Identification and characterization of macrophage inflammatory protein 2. Proc Natl Acad Sci. (1989) 86:612–6. doi: 10.1073/PNAS.86.2.612, PMID: 2643119 PMC286522

[50] Li Y Zheng Y Yang L Wang Q Bi E Li T. Chemokines CCL14 and CCL3 facilitate monocytes/macrophage infiltration in multiple myeloma bone marrow. Blood. (2014) 124:3380–0. doi: 10.1182/BLOOD.V124.21.3380.3380

[51] Kapellos TS Bonaguro L Gemünd I Reusch N Saglam A Hinkley ER. Human monocyte subsets and phenotypes in major chronic inflammatory diseases. Front Immunol. (2019) 10:2035/BIBTEX. doi: 10.3389/FIMMU.2019.02035/BIBTEX, PMID: 31543877 PMC6728754

[52] Zhao X Gu M Xu X Wen X Yang G Li L. CCL3/CCR1 mediates CD14+CD16– circulating monocyte recruitment in knee osteoarthritis progression. Osteoarthritis Cartilage. (2020) 28:613–25. doi: 10.1016/j.joca.2020.01.009, PMID: 32006659

[53] Menten P Wuyts A Van Damme J. Macrophage inflammatory protein-1. Cytokine Growth Factor Rev. (2002) 13:455–81. doi: 10.1016/S1359-6101(02)00045-X, PMID: 12401480

[54] Sindhu S Kochumon S Shenouda S Wilson A Al-Mulla F Ahmad R. The cooperative induction of CCL4 in human monocytic cells by TNF-α and palmitate requires myD88 and involves MAPK/NF-κB signaling pathways. Int J Mol Sci. (2019) 20:4658. doi: 10.3390/IJMS20184658, PMID: 31546972 PMC6770648

[55] Canning P Ruan Q Schwerd T Hrdinka M Maki JL Saleh D. Inflammatory signaling by NOD-RIPK2 is inhibited by clinically relevant type II kinase inhibitors. Chem Biol. (2015) 22:1174–84. doi: 10.1016/J.CHEMBIOL.2015.07.017, PMID: 26320862 PMC4579271

[56] Gudgeon J Marín-Rubio JL Trost M. The role of macrophage scavenger receptor 1 (MSR1) in inflammatory disorders and cancer. Front Immunol. (2022) 13:1012002/BIBTEX. doi: 10.3389/FIMMU.2022.1012002/BIBTEX, PMID: 36325338 PMC9618966

[57] Wang XF Zhou E Li DJ Mao CY He Q Zhang JF. VSTM1 regulates monocyte/macrophage function via the NF-κB signaling pathway. Open Med (Poland). (2021) 16:1513–24. doi: 10.1515/MED-2021-0353/MACHINEREADABLECITATION/RIS, PMID: 34712823 PMC8511964

[58] Reizis B Idoyaga J Dalod M Barrat F Naik S Trinchieri G. Reclassification of plasmacytoid dendritic cells as innate lymphocytes is premature. Nat Rev Immunol. (2023) 23:336–7. doi: 10.1038/s41577-023-00864-y, PMID: 36959479

[59] Ziegler-Heitbrock L Ohteki T Ginhoux F Shortman K Spits H. Reclassifying plasmacytoid dendritic cells as innate lymphocytes. Nat Rev Immunol. (2022) 23:1–2. doi: 10.1038/s41577-022-00806-0, PMID: 36380022

[60] Musumeci A Lutz K Winheim E Krug AB. What makes a PDC: Recent advances in understanding plasmacytoid DC development and heterogeneity. Front Immunol. (2019) 10:1222/BIBTEX. doi: 10.3389/FIMMU.2019.01222/BIBTEX, PMID: 31191558 PMC6548821

[61] Ye Y Gaugler B Mohty M Malard F. Plasmacytoid dendritic cell biology and its role in immune-mediated diseases. Clin Transl Immunol. (2020) 9:e1139. doi: 10.1002/CTI2.1139, PMID: 32489664 PMC7248678

[62] Bencze D Fekete T Pázmándi K. Type I interferon production of plasmacytoid dendritic cells under control. Int J Mol Sci. (2021) 22:4190. doi: 10.3390/IJMS22084190, PMID: 33919546 PMC8072550

[63] Asselin-Paturel C Trinchieri G. Production of type I interferons plasmacytoid dendritic cells and beyond. J Exp Med. (2005) 202:461–5. doi: 10.1084/JEM.20051395, PMID: 16103406 PMC2212850

[64] Colonna M Trinchieri G Liu YJ. Plasmacytoid dendritic cells in immunity. Nat Immunol. (2004) 5:1219–26. doi: 10.1038/ni1141, PMID: 15549123

[65] Getz GS. Bridging the innate and adaptive immune systems. J Lipid Res. (2005) 46:619–22. doi: 10.1194/jlr.E500002-JLR200, PMID: 15722562

[66] Huang X Dorta-Estremera S Yao Y Shen N Cao W. Predominant role of plasmacytoid dendritic cells in stimulating systemic autoimmunity. Front Immunol. (2015) 6:526/BIBTEX. doi: 10.3389/FIMMU.2015.00526/BIBTEX, PMID: 26528288 PMC4601279

[67] Vermi W Lonardi S Morassi M Rossini C Tardanico R Venturini M. Cutaneous distribution of plasmacytoid dendritic cells in lupus erythematosus. Selective tropism at the site of epithelial apoptotic damage. Immunobiology. (2009) 214:877–86. doi: 10.1016/J.IMBIO.2009.06.013, PMID: 19625100

[68] Werth VP Furie RA Romero-Diaz J Navarra S Kalunian K van Vollenhoven RF. Trial of anti-BDCA2 antibody litifilimab for cutaneous lupus erythematosus. New Engl J Med. (2022) 387:321–31. doi: 10.1056/NEJMOA2118024/SUPPL_FILE/NEJMOA2118024_DATA-SHARING.PDF 35939578

[69] Furie RA van Vollenhoven RF Kalunian K Navarra S Romero-Diaz J Werth VP. Trial of anti-BDCA2 antibody litifilimab for systemic lupus erythematosus. New Engl J Med. (2022) 387:894–904. doi: 10.1056/nejmoa2118025, PMID: 36069871

[70] Baumgart DC Metzke D Schmitz J Scheffold A Sturm A Wiedenmann B. Patients with active inflammatory bowel disease lack immature peripheral blood plasmacytoid and myeloid dendritic cells. Gut. (2005) 54:228–36. doi: 10.1136/gut.2004.040360, PMID: 15647187 PMC1774844

[71] Baumgart DC Metzke D Guckelberger O Pascher A Grötzinger C Przesdzing I. Aberrant plasmacytoid dendritic cell distribution and function in patients with Crohn’s disease and ulcerative colitis. Clin Exp Immunol. (2011) 166:46–54. doi: 10.1111/j.1365-2249.2011.04439.x, PMID: 21762123 PMC3193918

[72] Arimura K Takagi H Uto T Fukaya T Nakamura T Choijookhuu N. Crucial role of plasmacytoid dendritic cells in the development of acute colitis through the regulation of intestinal inflammation. Mucosal Immunol. (2016) 10:957–70. doi: 10.1038/mi.2016.96, PMID: 27848952

[73] Mizuno S Kanai T Mikami Y Sujino T Ono Y Hayashi A. CCR9+ plasmacytoid dendritic cells in the small intestine suppress development of intestinal inflammation in mice. Immunol Lett. (2012) 146:64–9. doi: 10.1016/J.IMLET.2012.05.001, PMID: 22626536

[74] Rahman T Brown AS Hartland EL Van Driel IR Fung KY. Plasmacytoid dendritic cells provide protection against bacterial-induced colitis. Front Immunol. (2019) 10:608/BIBTEX. doi: 10.3389/FIMMU.2019.00608/BIBTEX, PMID: 31024525 PMC6465541

[75] Sawai CM Serpas L Neto AG Jang G Rashidfarrokhi A Kolbeck R. Plasmacytoid dendritic cells are largely dispensable for the pathogenesis of experimental inflammatory bowel disease. Front Immunol. (2018) 9:2475/BIBTEX. doi: 10.3389/FIMMU.2018.02475/BIBTEX, PMID: 30410494 PMC6209677

[76] Barratt J Weitz I. Complement factor D as a strategic target for regulating the alternative complement pathway. Front Immunol. (2021) 12:712572/BIBTEX. doi: 10.3389/FIMMU.2021.712572/BIBTEX, PMID: 34566967 PMC8458797

[77] Bain CC Scott CL Uronen-Hansson H Gudjonsson S Jansson O Grip O. Resident and pro-inflammatory macrophages in the colon represent alternative context-dependent fates of the same Ly6Chi monocyte precursors. Mucosal Immunol. (2013) 6:498–510. doi: 10.1038/mi.2012.89, PMID: 22990622 PMC3629381

[78] Bain CC Schridde A. Origin, differentiation, and function of intestinal macrophages. Front Immunol. (2018) 9:2733. doi: 10.3389/fimmu.2018.02733, PMID: 30538701 PMC6277706

[79] Bain CC Mowat AM. The monocyte-macrophage axis in the intestine. Cell Immunol. (2014) 291:41–8. doi: 10.1016/j.cellimm.2014.03.012, PMID: 24726741 PMC4217150

[80] Turner D Ricciuto A Lewis A D’Amico F Dhaliwal J Griffiths AM. STRIDE-II: an update on the selecting therapeutic targets in inflammatory bowel disease (STRIDE) initiative of the international organization for the study of IBD (IOIBD): determining therapeutic goals for treat-to-target strategies in IBD. Gastroenterology. (2021) 160:1570–83. doi: 10.1053/J.GASTRO.2020.12.031/ATTACHMENT/F1BB64EB-27CC-4F26-9FE8-91734BCE443B/MMC3.PDF, PMID: 33359090

[81] Li Yim AYF. Towards the identification of Crohn’s disease-associated epigenetic biomarkers in leukocytes. A potential role in personalized diagnostics and treatment. Amsterdam: Universiteit van Amsterdam (2022) p. 1–298.

[82] Li Yim AYF Hageman IL Joustra V Elfiky A Ghiboub M Levin E. Single-cell characterization of peripheral blood mononuclear cells from Crohn’s disease patients on vedolizumab. medRxiv. (2023), 23291732. doi: 10.1101/2023.06.23.23291732

