# Supplementary material for: An exploratory single-cell analysis of peripheral blood mononuclear cells from vedolizumab-treated Crohn’s disease patients identifies response-associated differences among the plasmacytoid dendritic cells and classical monocytes
Source: Front Immunol. 2025 Aug 15;16:1551017. doi: 10.3389/fimmu.2025.1551017 (PMC12394049; doi:10.3389/fimmu.2025.1551017)
Supplement: Supplementary file 1 [file DataSheet1.docx]

Supplementary Material

# Supplementary Data

## Supplementary Materials and Methods

### General bioinformatic data analyses

Data was imported and analyzed using the R statistical environment (v4.2) (1) using several packages obtained from the Bioconductor (v3.16) (2) repository. The analytical workflow was orchestrated by Snakemake (v7.14.0) (3). Visualizations were typically created using the *tidyverse* (v1.3.1) (4), *ggplot2* (v3.4.2) (5), *ggrastr* (1.0.1), *ggrepel* (0.9.3), *cowplot* (v1.1.1), *viridis* (v0.6.3) (6), *pheatmap* (v1.0.12).

### Single-cell RNA-sequencing analysis

Samples were removed from the cryostat and thawed on ice. Thawed PBMCs were washed and then labelled using the BioLegend TotalSeq-B cell hashtag oligo (HTO) antibodies for multiplexing purposes per manufacturer’s protocol at 1 U per 1 million cells (7). An aliquot of the tagged PBMCs was assessed for viability using the Countess II FL Automated Cell Counter indicating that over 80% of the cells were viable. The resulting oligo-tagged cell suspensions were then mixed and distributed across 6 GEM-wells to be loaded onto the Chromium controller (10X Genomics) using 10X chemistry v3. Per well, 10,000 cells were loaded for a targeted recovery rate of up to 6,000 cells. Separate sequencing libraries were prepared for HTOs and the actual mRNA after size-selection and the libraries were sequenced on the Illumina HiSeq4000 in a 150 bp paired-ended fashion at the Core Facility Genomics, Amsterdam UMC. The mRNA libraries were sequenced on 150M reads per GEM-well, whereas the HTO libraries were sequenced to a depth of 50M reads per GEM-well. Raw reads were aligned and unique molecular identifier (UMI) count matrices were generated using Cellranger (v7.0.0). Subsequent import, sample-wise demultiplexing, processing, and analysis was done in Seurat (v4.3.0) (8). Cells that were identified as multiplets, based on the presence of an equal number of different HTOs, or that did obtain sufficient HTO signal were removed as they could not be assigned to a unique donor. Subsequent quality control included identifying dead cells based on mitochondrial read content (>75%) and a low number of unique genes, which were annotated accordingly (9). UMI counts were normalized using SCTransform (10), whereupon the cells were annotated by mapping our data onto a reference PBMC CITE-seq experiment of 162,000 annotated cells using a weighted nearest neighbor approach (11,12). A subsequent manual curation using canonical markers confirmed the identity of the different cell types. T cells were identified based on the expression of *CD3D*, *CD2*, *CD7*, and *IL7R*. Natural killer (NK) cells were identified based on the expression of *CD2*, *CD7*, *GNLY* and *NKG7*, while lacking *CD3D*, B-cells were identified based on the expression of *MS4A1* and *BANK1* positive. Monocytes were identified based on the expression of *CST3* and *CD14* or *FCGR3A*. Conventional dendritic cells (cDC) were identified on expression of *CD1C*, *CST3*, *FCER1A* and *HLA-DRA*. Smaller cell populations not belonging to the lineages were identified as well, namely the thrombocytes (*CST3* and *PPBP* positive), hematopoietic stem and progenitor cells (HSPCs; *CD34* positive), and erythroblasts (*HBA1*, *HBA2*, *HBQ1*, and *HBB* positive) (13). Differential abundance analyses were conducted by comparing the proportions using a t-test as implemented in the *speckle* (v0.99.7) (14) package where we omitted cell types that were represented by 10 cells or less. Differential expression analyses were performed by pseudobulk (15) approaches to account for cells coming from the same donor using the *DESeq2* (v1.36.0) (16) package. Gene set overrepresentation analyses were performed using Wald statistic as input for *fgsea* (v1.22.0) (17) against the Kyoto Encyclopedia for Genes and Genomes (KEGG) gene sets (18).

### Mass cytometry by time-of-flight analysis

An overview of the antibody mass cytometry panel (including metal tag and supplier) is listed in **Supplementary Table S1**. Cryopreserved PBMCs were thawed, washed with PBS, and resuspended in RPMI medium. Cellular viability was assessed through live/dead staining using 5μM Cisplatin in PBS at room temperature. Cisplatin signal was quenched by washing with Cell Staining Buffer (CSB; Fluidigm) after 5 minutes and washed away. Several targets in the panel lose their binding specificity after PFA fixation. Therefore the corresponding antibodies (listed in table S1) were incubated in the presence of Human TruStain FcX™ Fc Receptor Blocking Solution (Biolegend) at room temperature for 30 minutes and washed away. Afterwards cells were fixed with 1.6% PFA and labeled using the Cell-ID 20-Plex Pd Barcoding Kit (Fluidigm) for multiplexing purposes per manufacturer’s protocol. Pooled cells were then stained for remaining cell-surface targets. Antibody concentrations were optimized for staining 3M cells per 100 μL of CSB for 30 minutes at room temperature. For intracellular staining, cells were washed and incubated with antibodies for intracellular markers (CES1 and CTLA4). CES1 lacked a metal reporter but was the only rabbit anti-human antibody, so a goat anti-rabbit antibody coupled to 175Lu was used as a secondary staining for CES1. After washing with CSB, antibodies were again fixed with 1.6% PFA, washed and incubated overnight with ^191/193^Ir DNA intercalator (1:4000) diluted in Fix-and-Perm Buffer (Fluidigm). Cells were subsequently washed before data acquisition was performed on the CyTOF3-Helios (Fluidigm). After data acquisition, raw .FCS files were imported in R. Expression values were arcsinh-transformed with cofactor 5. Signal intensities and sample acquisition rates were reviewed for stability over time and events gated based on the condition that the flow was stable, excluding calibration beads, and within the 90th percentile of all Gaussian parameters. Resulting singlets were selected for CD45^+^ signal. Cells were clustered in an unsupervised manner using the FlowSOM-package, where initial SOM-clustering was set to 300 clusters, using markers listed in **Supplementary Table S1**. The 300 clusters were subsequently manually metaclustered according to their phenotypic lineages, whereafter cells were annotated. UMAP dimensionality reduction was performed using the *uwot* (0.1.14). Cells were subsampled to 16,000 cells to approximately match the number of cells identified through scRNAseq.

### **Flow cytometry analysis**

This protocol is identical to the one implemented in Elfiky *et al.* (19). An overview of the antibody flow cytometry panel (including metal tag and supplier) is listed in **Supplementary Table S2**. Cryopreserved PBMCs were thawed, washed with PBS and stained for a live/dead cell viability marker (LifeScience, Amsterdam, the Netherlands). Cells were stained for surface markers in FACS buffer (0.5% BSA, 0.01% NaN3 in PBS) using the following antibodies: CD11c-PerCP Cy5.5 (clone: S-HCL-3, BioLegend), HLA-DR-Alexa Fluor 700 (clone: LN3, eBioscience), CD123-FITC (clone: 6H6, BioLegend), CD1C-PE-Cy7 (clone: L161, BioLegend), pan-lineage (CD3/CD19/CD20/CD56)-APC (clones: UCHT1;HIB19;2H7;5.1H11, BioLegend), CD14-BD Horizon V500 (clone: M5E2, Becton Dickinson) and CD16-PE (clone: 3G8, Becton Dickinson). Doublets were excluded and live single cells identified using the forward scatter height (FSC-H) versus the forward scatter area (FSC-A) and the side scatter height (SSC-H) versus side scatter area (SSC-A). Live cells were identified using the live/dead marker. Classical monocytes were defined as Lin^-^HLA−DR^+^CD14^++^CD16^-^, intermediate monocytes as Lin^-^HLA−DR^+^CD14^++^CD16^+^, and non-classical monocytes as Lin^-^HLA-DR^+^CD14^+^CD16^+^. Conventional dendritic cells (cDCs) were defined as Lin^-^HLA-DR^+^CD11c^+^CD1c^+^ and plasmacytoid DCs (pDCs) as Lin^-^HLA-DR^+^CD11c^-^CD123^+^. Fluorescence minus one (FMO) was used for gating and median fluorescence intensity was determined to quantify cell surface expression.

### RNA-sequencing analysis classical monocytes

PBMCs were subjected to flow cytometric sorting where classical monocytes were identified as Lin^-^HLA−DR^+^CD14^++^CD16^-^. Due to low input material, classical monocytes mRNA was converted into cDNA using the Ovation RNA-seq System V2 kit (NuGEN; Agilent, Santa Clara, United States), whereupon sequencing libraries were prepared using the Ovation Ultralow System V2 kit (NuGEN; Agilent, Santa Clara, United States) and thereafter sequenced in a 150 bp paired-ended fashion on the Illumina NovaSeq6000 to a depth of 40 million reads at the Amsterdam UMC Core Facility Genomics. Quality control of the raw reads was done using FastQC (v0.11.8) (20) and MultiQC (v1.0) (21). Raw reads were aligned to the human genome (GRCh38) using STAR (v2.7.0) and annotated using the Ensembl (v95) annotation (22). Post-alignment processing was performed through SAMtools (v1.9) (23), after which reads were counted using the featureCounts function found in the Subread package (v1.6.3) (24). Differential expression (DE) analysis was performed using *DESeq2* (v1.36.0) (16).

# Supplementary Figures and Tables

## Supplementary Figures


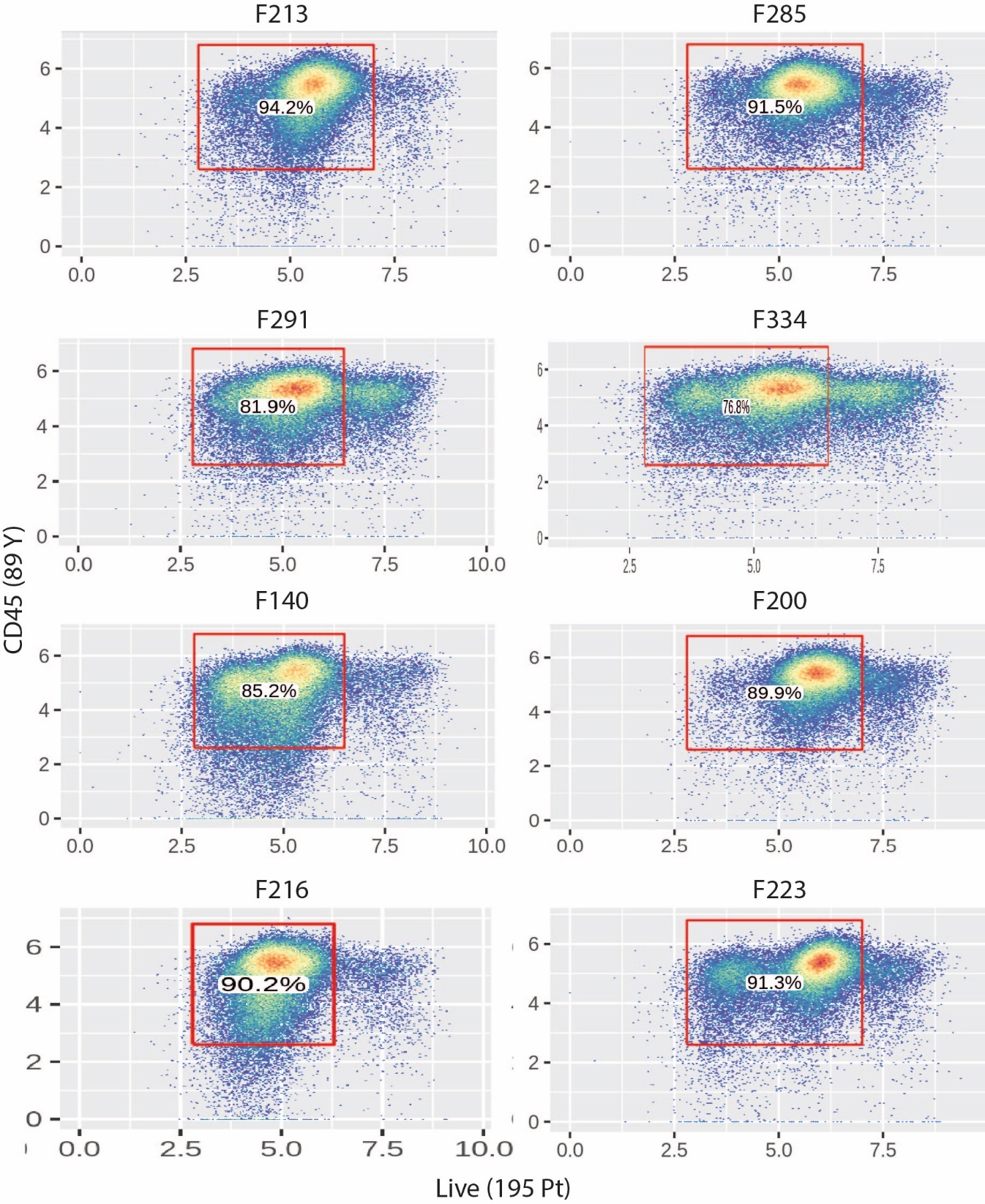


**Supplemental Figure 1.** Mass cytometric scattered density plot of the live/dead stain (X-axis) and CD45 (Y-axis) of PBMCs from the patient material included in the study. Each plot represents an individual patient sample and the red box represents the cells included for subsequent analyses.


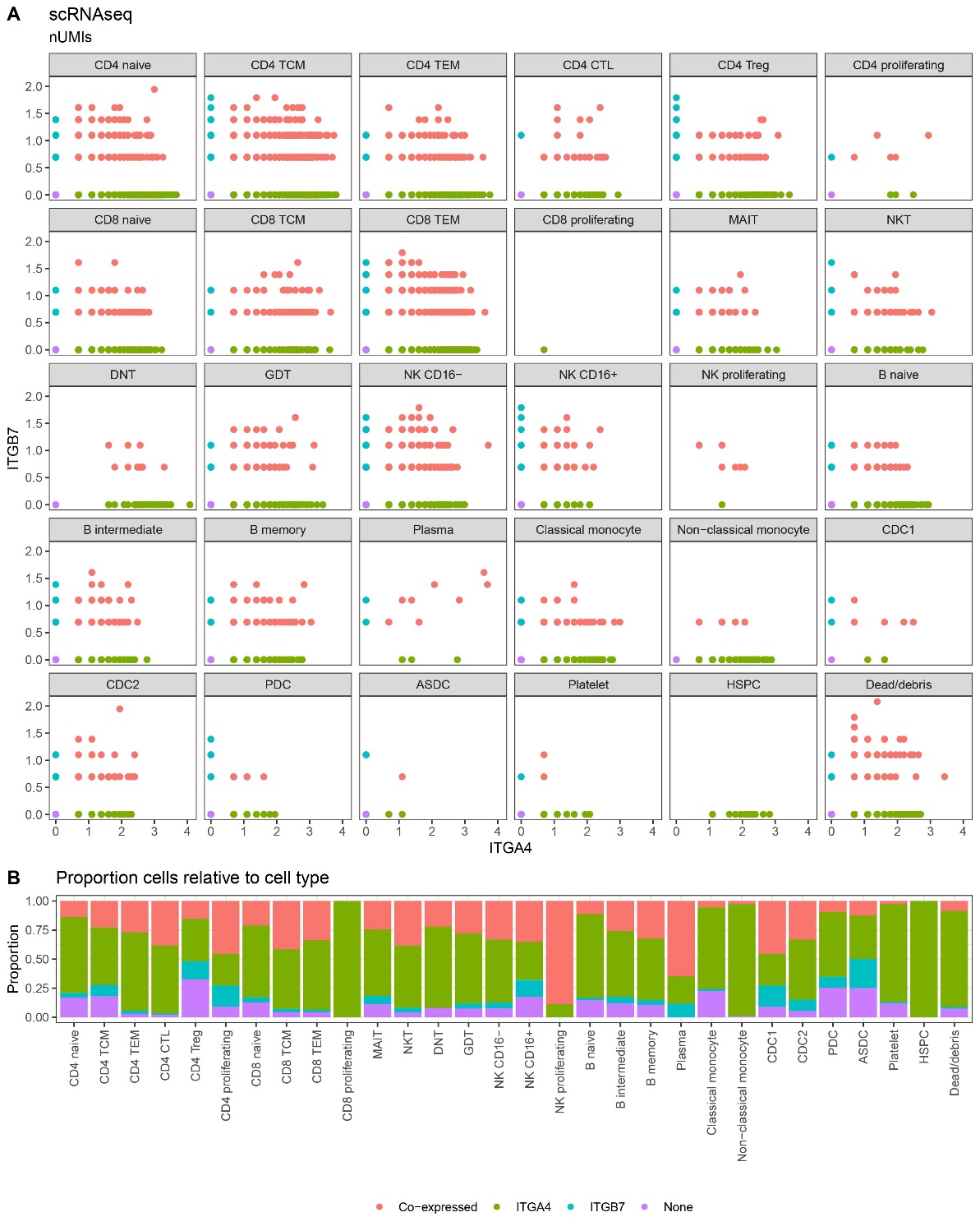


**Supplemental Figure 2.** Gene co-expression of ITGA4 and ITGB7 obtained through scRNAseq. (A) Scatterplot visualizing gene expression in normalized UMIs (nUMIs) of *ITGA4* and *ITGB7* on the X- and Y-axis, respectively, per cell. Colors represent cells that display measurable expression of *ITGA4* only (green), *ITGB7* only (blue), *ITGA4* and *ITGB7* (red), or none (purple). B) Stacked barplots depicting the proportion cells that display measurable expression of *ITGA4* only (green), *ITGB7* only (blue), *ITGA4* and *ITGB7* (red), or none (purple) per cell type.


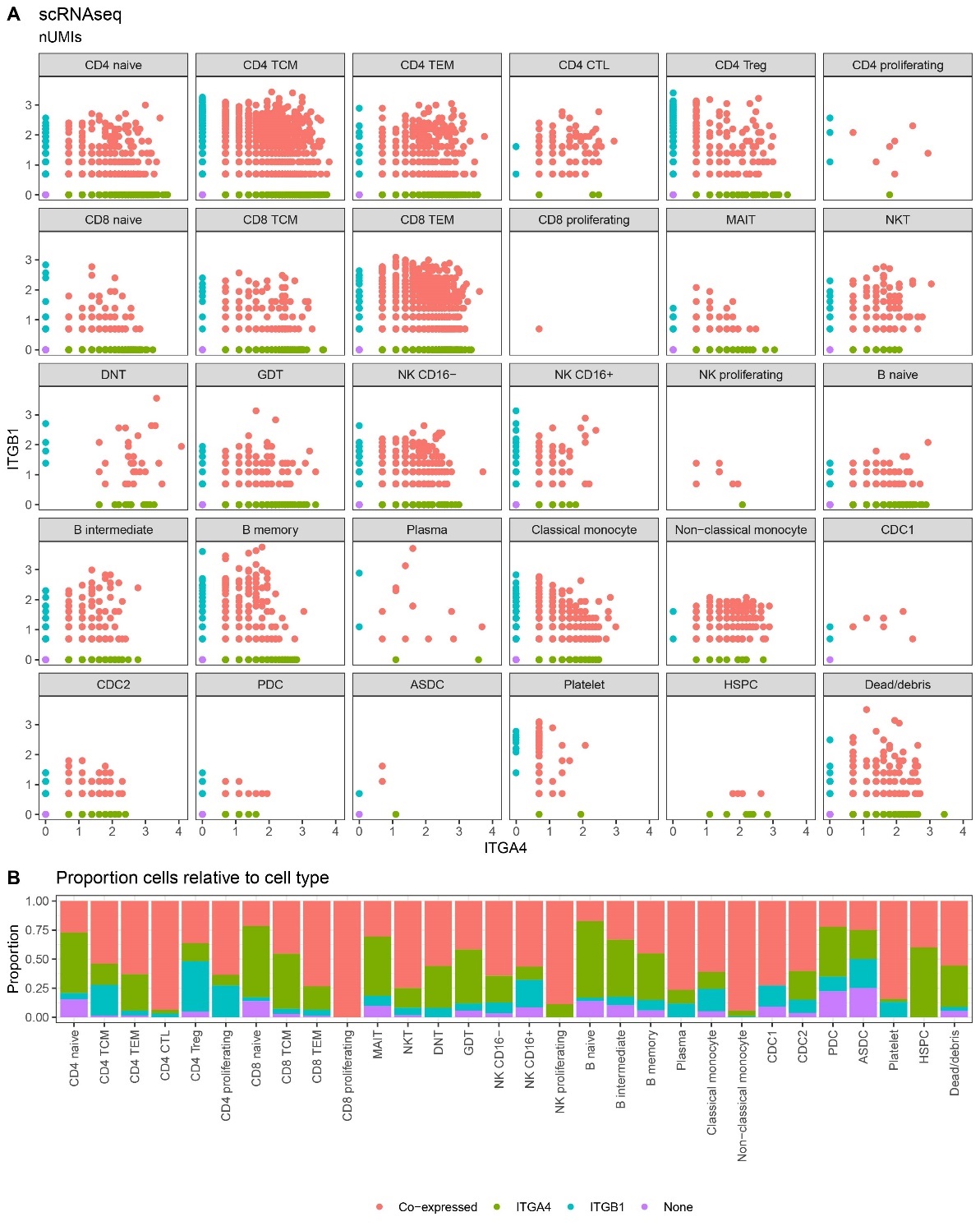


**Supplemental Figure 3.** Gene co-expression of ITGA4 and ITGB1 obtained through scRNAseq. (A) Scatterplot visualizing gene expression in normalized UMIs (nUMIs) of *ITGA4* and *ITGB1* on the X- and Y-axis, respectively, per cell. Colors represent cells that display measurable expression of *ITGA4* only (green), *ITGB1* only (blue), *ITGA4* and *ITGB7* (red), or none (purple). B) Stacked barplots depicting the proportion cells that display measurable expression of *ITGA4* only (green), *ITGB7* only (blue), *ITGA4* and *ITG1* (red), or none (purple) per cell type.


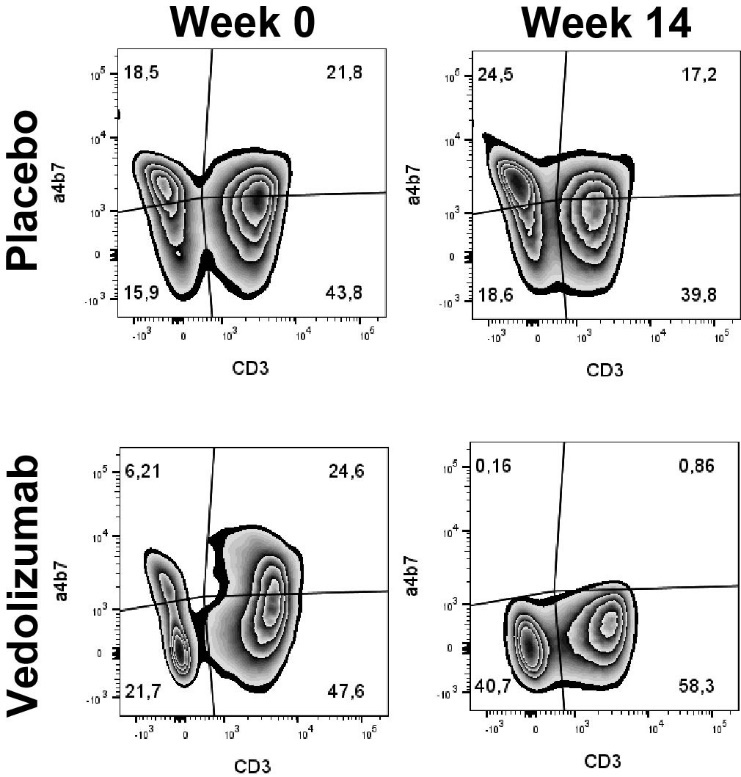


**Supplemental Figure 4.** Flow cytometric scattered contour plot of CD3 (X-axis) and vedolizumab (Y-axis) of PBMCs obtained from UC patients with pouchitis treated with either placebo or vedolizumab before (week 0) or during (week 14) treatment.

## Supplementary Tables

**Supplemental Table 1. Marker panel mass cytometry**. The cell-surface exposed markers assayed in the mass cytometry experiment annotated by the metal, target protein, alternative names, uniport identifier, and notes.

**Supplemental Table 2. Marker panel flow cytometry**. The cell-surface exposed markers assayed in the flow cytometry experiment alongside the antibody and clone.

**Supplemental** **Table 3. PBMC differential abundance analysis.** Results of the differential abundance analysis on the major lineages as conducted using the propeller function in speckle. Columns represent the cell type, the mean proportion for all samples (“BaselineProp.Freq”), non-responders only (“PropMean.Non.responder”), and responders only (“PropMean.Responder”), the ratio responder/non-responder (“PropRatio”), the associated T statistic (“Tstatistic”) as well as the *p­*-values (“P.Value” and “FDR”). Tabs separate the results from scRNAseq and CyTOF analyses.

**Supplemental Table 4. T cell scRNAseq pseudobulk differential gene expression analysis.** Results of the differential expression analysis performed on PDCs using DESeq2. Columns represent the gene, the average expression (“baseMean”), the log_2_(fold change) (“log2FoldChange”), the associated standard error (“lfcSE”), the Wald statistic (“stat”) as well as the *p­*-values (“pvalue” and “padj”). Each tab represents a different T cell subset.

**Supplemental Table 5. pDC scRNAseq pseudobulk differential gene expression analysis.** Results of the differential expression analysis performed on PDCs using DESeq2. Columns represent the gene, the average expression (“baseMean”), the log_2_(fold change) (“log2FoldChange”), the associated standard error (“lfcSE”), the Wald statistic (“stat”) as well as the *p­*-values (“pvalue” and “padj”).

**Supplemental Table 6. Classical monocytes scRNAseq pseudobulk differential gene expression analysis.** Results of the differential expression analysis performed on classical monocytes using DESeq2. Columns represent the gene, the average expression (“baseMean”), the log_2_(fold change) (“log2FoldChange”), the associated standard error (“lfcSE”), the Wald statistic (“stat”) as well as the *p­*-values (“pvalue” and “padj”).

**Supplemental Table 7. Classical monocytes bulk RNAseq gene expression analysis.** Results of the differential expression analysis performed on classical monocytes using DESeq2. Columns represent the gene and Ensembl ID, the average expression (“baseMean”), the log_2_(fold change) (“log2FoldChange”), the associated standard error (“lfcSE”), the Wald statistic (“stat”) as well as the *p­*-values (“pvalue” and “padj”).

**Supplemental Table 8. Classical monocytes scRNAseq pseudobulk KEGG gene set enrichment analysis**. Gene set enrichment analysis as performed by fgsea. Columns represent the gene set (“Pathway”), the *p-*values (“pvalue” and “FDR”), the log_2_ standard error (“log2err”), the enrichment score (“ES”), the normalized enrichment score (“NES”), and the total number of genes in the gene set (“size”).

# References

1. R Development Core Team. *R: A language and environment for statistical computing*. Vienna, Austria: R Foundation for Statistical Computing. (2008). http://www.r-project.org/

2. Gentleman RC, Carey VJ, Bates DM, Bolstad B, Dettling M, Dudoit S, Ellis B, Gautier L, Ge Y, Gentry J, et al. Bioconductor: open software development for computational biology and bioinformatics. *Genome Biol* (2004) 5:R80. doi: 10.1186/gb-2004-5-10-r80

3. Köster J, Mölder F, Jablonski KP, Letcher B, Hall MB, Tomkins-Tinch CH, Sochat V, Forster J, Lee S, Twardziok SO, et al. Sustainable data analysis with Snakemake. *F1000Research 2021 10:33* (2021) 10:33. doi: 10.12688/f1000research.29032.2

4. Wickham H, Averick M, Bryan J, Chang W, McGowan LD, François R, Grolemund G, Hayes A, Henry L, Kuhn JH and M, et al. Welcome to the tidyverse. *J Open Source Softw* (2019) 4:1686. doi: 10.21105/joss.01686

5. Wickham H. *ggplot2: Elegant Graphics for Data Analysis*. New York, NY: Springer-Verlag New York. (2009). http://ggplot2.org

6. Garnier, Simon, Ross, Noam, Robert R and, Camargo, Pedro A, Sciaini, Marco, Scherer, et al. viridis(Lite) - Colorblind-Friendly Color Maps for R. (2023) doi: 10.5281/zenodo.4679424

7. Stoeckius M, Zheng S, Houck-Loomis B, Hao S, Yeung BZ, Mauck WM, Smibert P, Satija R. Cell Hashing with barcoded antibodies enables multiplexing and doublet detection for single cell genomics. *Genome Biol* (2018) 19:224. doi: 10.1186/s13059-018-1603-1

8. Butler A, Hoffman P, Smibert P, Papalexi E, Satija R. Integrating single-cell transcriptomic data across different conditions, technologies, and species. *Nat Biotechnol* (2018) 36:411–420. doi: 10.1038/nbt.4096

9. Luecken MD, Theis FJ. Current best practices in single‐cell RNA‐seq analysis: a tutorial. *Mol Syst Biol* (2019) 15:e8746. doi: 10.15252/msb.20188746

10. Hafemeister C, Satija R. Normalization and variance stabilization of single-cell RNA-seq data using regularized negative binomial regression. *Genome Biol* (2019) 20:296. doi: 10.1101/576827

11. Stuart T, Butler A, Hoffman P, Hafemeister C, Papalexi E, Mauck  3rd WM, Hao Y, Stoeckius M, Smibert P, Satija R. Comprehensive Integration of Single-Cell Data. *Cell* (2019) 177:1888-1902.e21. doi: 10.1016/j.cell.2019.05.031

12. Hao Y, Hao S, Andersen-Nissen E, Mauck WM, Zheng S, Butler A, Lee MJ, Wilk AJ, Darby C, Zager M, et al. Integrated analysis of multimodal single-cell data. *Cell* (2021) 184:3573-3587.e29. doi: 10.1016/J.CELL.2021.04.048

13. Franzén O, Gan LM, Björkegren JLM. PanglaoDB: A web server for exploration of mouse and human single-cell RNA sequencing data. *Database* (2019) 2019: doi: 10.1093/database/baz046

14. Phipson B, Sim CB, Porrello ER, Hewitt AW, Powell J, Oshlack A. propeller: testing for differences in cell type proportions in single cell data. *Bioinformatics* (2022) 38:4720–4726. doi: 10.1093/BIOINFORMATICS/BTAC582

15. Squair JW, Gautier M, Kathe C, Anderson MA, James ND, Hutson TH, Hudelle R, Qaiser T, Matson KJE, Barraud Q, et al. Confronting false discoveries in single-cell differential expression. *Nature Communications 2021 12:1* (2021) 12:1–15. doi: 10.1038/s41467-021-25960-2

16. Love MI, Huber W, Anders S. Moderated estimation of fold change and dispersion for RNA-seq data with DESeq2. *Genome Biol* (2014) 15:550. doi: 10.1186/s13059-014-0550-8

17. Sergushichev AA. An algorithm for fast preranked gene set enrichment analysis using cumulative statistic calculation. *bioRxiv* (2016)060012. doi: 10.1101/060012

18. Kanehisa M, Sato Y, Kawashima M, Furumichi M, Tanabe M. KEGG as a reference resource for gene and protein annotation. *Nucleic Acids Res* (2016) 44:D457–D462. doi: 10.1093/nar/gkv1070

19. Elfiky AMI, Hageman IL, Becker MAJ, Verhoeff J, Li Yim AYF, Joustra VW, Mulders L, Fung I, Rioja I, Prinjha RK, et al. A BET Protein Inhibitor Targeting Mononuclear Myeloid Cells Affects Specific Inflammatory Mediators and Pathways in Crohn’s Disease. *Cells* (2022) 11: doi: 10.3390/cells11182846

20. Andrews S. FastQC: a quality control tool for high throughput sequence data. http://www.bioinformatics.babraham.ac.uk/projects/fastqc

21. Ewels P, Magnusson M, Lundin S, Kaller M. MultiQC: summarize analysis results for multiple tools and samples in a single report. *Bioinformatics* (2016) 32:3047–3048. doi: 10.1093/bioinformatics/btw354

22. Cunningham F, Allen JE, Allen J, Alvarez-Jarreta J, Amode MR, Armean IM, Austine-Orimoloye O, Azov AG, Barnes I, Bennett R, et al. Ensembl 2022. *Nucleic Acids Res* (2022) 50:D988–D995. doi: 10.1093/NAR/GKAB1049

23. Li H, Handsaker B, Wysoker A, Fennell T, Ruan J, Homer N, Marth G, Abecasis G, Durbin R, Genome Project Data Processing S. The Sequence Alignment/Map format and SAMtools. *Bioinformatics* (2009) 25:2078–2079. doi: 10.1093/bioinformatics/btp352

24. Liao Y, Smyth GK, Shi W. The Subread aligner: fast, accurate and scalable read mapping by seed-and-vote. *Nucleic Acids Res* (2013) 41:e108. doi: 10.1093/nar/gkt214
